# Sox2 Is Essential for Formation of Trophectoderm in the Preimplantation Embryo

**DOI:** 10.1371/journal.pone.0013952

**Published:** 2010-11-12

**Authors:** Maria Keramari, Janet Razavi, Karen A. Ingman, Christoph Patsch, Frank Edenhofer, Christopher M. Ward, Susan J. Kimber

**Affiliations:** 1 Faculty of Life Sciences, University of Manchester, Manchester, United Kingdom; 2 Stem Cell Engineering Group, Institute of Reconstructive Neurobiology, University of Bonn - Life & Brain Center and Hertie Foundation, Bonn, Germany; 3 Faculty of Medical and Human Sciences, University of Manchester, Manchester, United Kingdom; University of Southern California, United States of America

## Abstract

**Background:**

In preimplantation mammalian development the transcription factor Sox2 (SRY-related HMG-box gene 2) forms a complex with Oct4 and functions in maintenance of self-renewal of the pluripotent inner cell mass (ICM). Previously it was shown that *Sox2*−/− embryos die soon after implantation. However, maternal *Sox2* transcripts may mask an earlier phenotype. We investigated whether Sox2 is involved in controlling cell fate decisions at an earlier stage.

**Methods and Findings:**

We addressed the question of an earlier role for Sox2 using RNAi, which removes both maternal and embryonic *Sox2* mRNA present during the preimplantation period. By depleting both maternal and embryonic *Sox2* mRNA at the 2-cell stage and monitoring embryo development in vitro we show that, in the absence of *Sox2*, embryos arrest at the morula stage and fail to form trophectoderm (TE) or cavitate. Following knock-down of *Sox2* via three different short interfering RNA (siRNA) constructs in 2-cell stage mouse embryos, we have shown that the majority of embryos (76%) arrest at the morula stage or slightly earlier and only 18.7–21% form blastocysts compared to 76.2–83% in control groups. In *Sox2* siRNA-treated embryos expression of pluripotency associated markers Oct4 and Nanog remained unaffected, whereas TE associated markers Tead4, Yap, Cdx2, Eomes, Fgfr2, as well as Fgf4, were downregulated in the absence of Sox2. Apoptosis was also increased in *Sox2* knock-down embryos. Rescue experiments using cell-permeant Sox2 protein resulted in increased blastocyst formation from 18.7% to 62.6% and restoration of Sox2, Oct4, Cdx2 and Yap protein levels in the rescued *Sox2-*siRNA blastocysts.

**Conclusion and Significance:**

We conclude that the first essential function of Sox2 in the preimplantation mouse embryo is to facilitate establishment of the trophectoderm lineage. Our findings provide a novel insight into the first differentiation event within the preimplantation embryo, namely the segregation of the ICM and TE lineages.

## Introduction

*Sox* genes are expressed throughout embryogenesis and encode a subclass of high mobility group (HMG) box proteins driving cell fate decisions by acting as transcription factors and architectural components of chromatin [1], [2]. Sox2 is developmentally regulated [3] and is detected in the inner cell mass (ICM) of the murine blastocyst [4] and subsequently in primitive ectoderm, extraembryonic ectoderm [4] and the developing nervous system [5]. Expression of Sox2 is observed in mouse and human eye lens [6]; in humans, heterozygous loss-of-SOX2 function causes several defects including bilateral anophthalmia [7], and defects in the hypothalamo-pituitary-gonadal axis [8]. It is essential for inner ear sensory organ [9] and taste bud sensory cell [10] development. Sox2 acts cooperatively with the pluripotency factor Oct4 at promoters activating transcription of *Fgf4*, *Utf1* and *Fbx15* genes [11]–[13], and interacts with Nanog in regulating transcription of *Rex1* [14]. It has been reported that the crucial role for *Sox2* in mouse embryonic stem (ES) cells is to maintain them in a pluripotent state by preserving the required level of *Oct4* expression [15]. Furthermore, mouse embryonic and adult fibroblasts can be induced to a pluripotent state in vitro through ectopic expression of the transcription factors *Sox2*, *Oct4*, *c-Myc* and *Klf4* [16], [17], [18].

*Sox2* expression is essential during embryogenesis; *Sox2* homozygous null embryos die soon after implantation [4] and Sox2 is the earliest marker of inner cells prior to ICM formation [19]. Furthermore, Sox2 in association with the POU domain transcription factor Oct4 and homeobox transcription factor Nanog form a regulatory core, which maintains self-renewal of the pluripotent ICM in the embryo and ES cells [4], [15], [20]–[21], and is unique to mammals [22]. A contiguous pair of highly evolutionarily conserved Oct- and Sox-binding sites is essential for activating expression of genes specific to the pluripotent state in ES cells [23]. *Sox2* transcription is regulated by an enhancer containing this composite Sox-Oct cis-regulatory element that Sox2 and Oct4 bind synergistically [24]. This element also occurs within the proximal promoter of *Nanog* [21], essential for retaining pluripotency [25], [26]. The Sox2-Oct4-Nanog regulatory complex controls expression of pluripotency genes through feed-forward loops [22] including these three genes in an autoregulatory circuit [27]. As well as activating target genes essential for self-renewal, the Sox2-Oct4-Nanog complex represses genes initiating differentiation [28].

Blastocyst formation coincides with demarcation of the first two lineages in the mammalian preimplantation embryo: the ICM that gives rise to the embryo proper, extraembryonic endoderm and mesoderm, and the trophectoderm (TE) that generates the placenta [29], [30]. ES cells are derived from the ICM/epiblast population of the blastocyst [31]–[34]. Although this is a transitory cell-population in the embryo, cultured ES cells can undergo unlimited self-renewal and are pluripotent, giving rise to all embryonic cell types. At the late blastocyst-stage, three distinct cell lineages are observed: the epiblast, the primitive endoderm (PE) and the trophectoderm [35]. Key binary switches in the mouse blastocyst are regulated by pairs of transcription factors [36], [37] that govern cell fate decisions. Oct4, Sox2 and Nanog are fundamental regulators maintaining undifferentiated ES cell and epiblast fate [4], [20], [25], [26], while caudal-type homeobox transcription factor Cdx2 regulates TE gene expression and maintenance in the mouse blastocyst, repressing Oct4 and Nanog in the TE [38]. Recently Gata3 has been demonstrated to regulate trophoblast development in parallel to Cdx2, and both genes are dependent on a third gene, Tead4 [39], [40]. Gata6 regulates PE genes [41] antagonising Nanog within the mouse blastocyst [42]. In addition, the Spalt transcription factor Sall4 has been reported to regulate transcription of *Pou5f1* (encoding *Oct4*) and thereby formation of the ICM-derived lineages, namely the epiblast and the PE [43], [44].

*Fgf4*, regulated by the Sox2-Oct4 synergy [13], is expressed in the ICM [45] and has a role in ICM maintenance as well as trophoblast stem cell proliferation [37]. It has been suggested that Fgf4 could have a paracrine effect in inducing polar TE, adjacent to the ICM [46]. Fgf4 interacts with the receptor Fgfr2 [47] which is expressed by the TE and extraembryonic ectoderm [48]. Cdx2 and Eomesodermin (Eomes), a T-box transcription factor expressed in TE and responsible for its proliferation [38], [49], have been suggested as potential down-stream targets of Fgf4 signalling [46]. Regulation of these gene families have also been associated with Fgf4 signalling in *Danio rerio* and *Xenopus laevis* [50], [51].

Homozygous *Sox2* knock-out embryos die soon after implantation [4]. Implantation sites lacked the epiblast and extraembryonic ectoderm components although trophoblast giant cells could be distinguished. In culture, *Sox2* null embryos formed abnormal outgrowths lacking an ICM. *Sox2* homozygous mutant embryos could be rescued by wild-type ES cells suggesting cell autonomous function. However by targeted deletion of the *Sox2* gene, only mRNA from *Sox2* zygotic transcription can be eliminated; any persisting maternal *Sox2* mRNA or protein in the preimplantation embryo might fulfill earlier essential roles. We have addressed the question of an earlier role for Sox2 in controlling cell fate decisions using RNAi, which removes both maternal and embryonic *Sox2* mRNA present during the preimplantation period. By depleting both sources of *Sox2* mRNA at the 2-cell stage and monitoring embryo development in vitro, we showed that in the absence of *Sox2*, embryos arrest at the morula stage, and they fail to form TE or to cavitate. Furthermore, by performing rescue experiments using cell-permeant Sox2 protein, we observed reversal of the *Sox2-*siRNA phenotype, as demonstrated by blastocyst formation and expression of ICM, as well as TE markers.

## Results

### Sox2 expression in mouse preimplantation development

**Figure 1:**
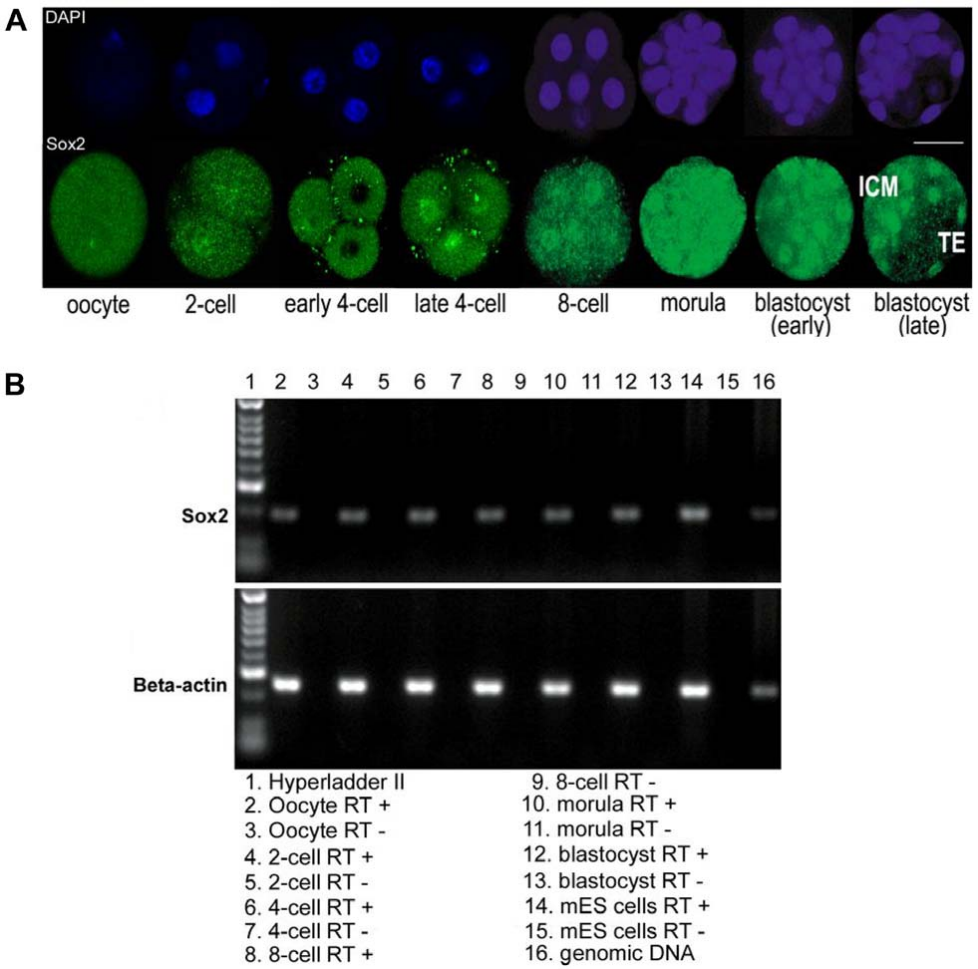
Sox2 (protein and mRNA) expression in mouse preimplantation development. Expression of Sox2 was detected at all stages (oocyte to blastocyst) of mouse preimplantation development by immunocytochemistry and RT-PCR. (**A**) Developmental stages of preimplantation mouse embryos immunostained for Sox2 (Abcam): single optical sections of confocal Z-series. Bar: 50 µm. (**B**) RT-PCR for *Sox2* and *β-actin* in mouse preimplantation embryos, 40 cycles. Lane 1: Hyperladder IV (Bioline); 2: oocyte; 4: 2-cell; 6: 4-cell; 8: 8-cell; 10: morula; 12: blastocyst; 14: mES cells (line *E14*); 16: genomic DNA (Bioline). Lanes (3, 5, 7, 9, 11, 13, 15): RT –ve controls for each developmental stage.

Temporal and spatial expression of Sox2 protein was examined in preimplantation mouse embryos by immunofluorescence. Sox2 protein was found in oocytes and 2-cell stage embryos; fluorescence intensity increased from the 4-8-cell stage to the morula stage, peaking at the blastocyst, where it was present in both TE and ICM. Sox2 protein was located in nuclei in cleavage stage embryos, although nuclear staining at the 2-cell stage was weak and cytoplasmic staining was observed. However in some 4-cell embryos, Sox2 was localized in nuclei in all blastomeres, while in others, nuclear localization was observed in half of the blastomeres, while still in others Sox2 staining was detected only in the cytoplasm and not in nuclei (Figure 1A). In blastocysts it was observed in some nuclei and cytoplasm of TE as well as in all ICM nuclei (Figure 1A). Pluripotent mouse ES cells and ES cells induced to form neural lineages were used as positive controls for Sox2 immunostaining (Figure S1A). Western blotting with the Sox2 antibody on protein extracts from mES cells revealed a single band of 37 kDa, the expected size for Sox2 protein (Figure S1B). To confirm these results, *Sox2* transcripts were also detected by RT-PCR at the oocyte, 2-cell, 4-cell, 8-cell, morula and blastocyst stages (Figure 1B).

### Role of Sox2 in mouse preimplantation development

*Sox2* was transiently knocked down in 2-cell *MF1*x*CD1* embryos with three different siRNA duplexes using electroporation. Duplexes for an irrelevant protein, Firefly Luciferase, and Galanin, a protein expressed throughout preimplantation mouse development (J. Razavi, D. Brison and S. Kimber, unpublished) were used as controls. Electroporation efficiency was 100%, as determined by rhodamine fluorescence detected immediately after electroporation and penetration of the siRNA duplexes into both cells of the 2-cell mouse embryos, which were compared to mock-electroporated embryos to exclude any autofluorescence effects. UV visualised embryos were not cultured further.

**Figure 2:**
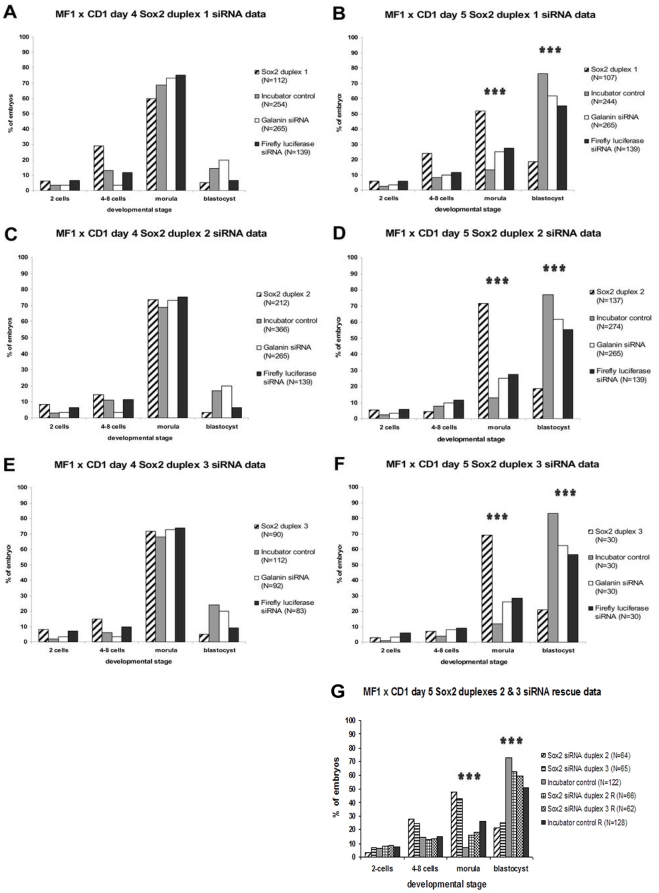
Development of *MF1*x*CD1* (A,B,C,D,E,F) embryos in culture after RNAi, as well as rescue phenotype of *MF1XCD1* (G) embryos. (**A,B**) *Sox2*-duplex-1-siRNA embryos (hatched, N = 112 day 4, N = 107 day 5) were compared with incubator-control (grey, N = 254 day 4, N = 244 day 5), *Galanin-*siRNA (white, N = 265) and *FFL*-siRNA (black, N = 139) embryos. Most day 4 (A) embryos formed morulae (16–32 cells), but 29% of the *Sox2*-duplex-1-siRNA embryos remained at the 4-8-cell stage. On day 5 (B), while 76.2% of incubator-control embryos formed blastocysts, only 18.7% of the *Sox2-*duplex-1-siRNA embryos reached blastocyst stage. (**C,D**) *Sox2*-duplex-2-siRNA embryos (hatched, N = 212 day 4, N = 137 day 5) were compared with incubator-control (grey, N = 366 day 4, N = 274 day 5), *Galanin-*siRNA (white, N = 265) and *FFL*-siRNA (black, N = 139) embryos. Most day 4 (C) embryos formed morulae, but 14.4% of the *Sox2-*duplex-2-siRNA embryos remained at the 4-8-cell stage. Only 18.7% of the day 5 (D) *Sox2*-duplex-2-siRNA embryos formed blastocysts. (**E,F**) *Sox2*-duplex-3-siRNA embryos (hatched, N = 90 day 4, N = 30 day 5) were compared with incubator-control (grey, N = 112 day 4, N = 30 day 5), *Galanin*-siRNA (white, N = 92 day 4, N = 30 day 5) and *FFL*-siRNA (black, N = 83 day 4, N = 30 day 5) embryos. Most day 4 (E) embryos formed morulae, while 15% of the *Sox2*-duplex-3-siRNA embryos remained at the 4-8-cell stage. Only 21% of the day 5 (F) *Sox2*-duplex-3-siRNA embryos developed to blastocysts. (**G**) Rescue phenotype at day 5 of development of *MF1xCD1* embryos. Only 21.1% of *Sox2*-duplex-2-siRNA embryos (N = 64) and 25.4% of *Sox2*-duplex-3-siRNA embryos (N = 65) formed blastocysts. After treatment with cell-permeant Sox2 protein, 62.6% of *Sox2*-duplex-2-siRNA R embryos (N = 66) formed blastocysts and 59.7% of the *Sox2*-duplex-3-siRNA R embryos (N = 62) reached blastocyst stage. 72.75% of untreated incubator control embryos (N = 122) formed blastocysts but treatment with cell-permeant Sox2 protein (N = 128) reduced this to 50.9%. In all cases, chi-square tests revealed significant differences (p<0.0001) between the % of *Sox2*-siRNA, control and R morulae, as well as between the % of *Sox2*-siRNA, control and R blastocysts.

**Figure 3:**
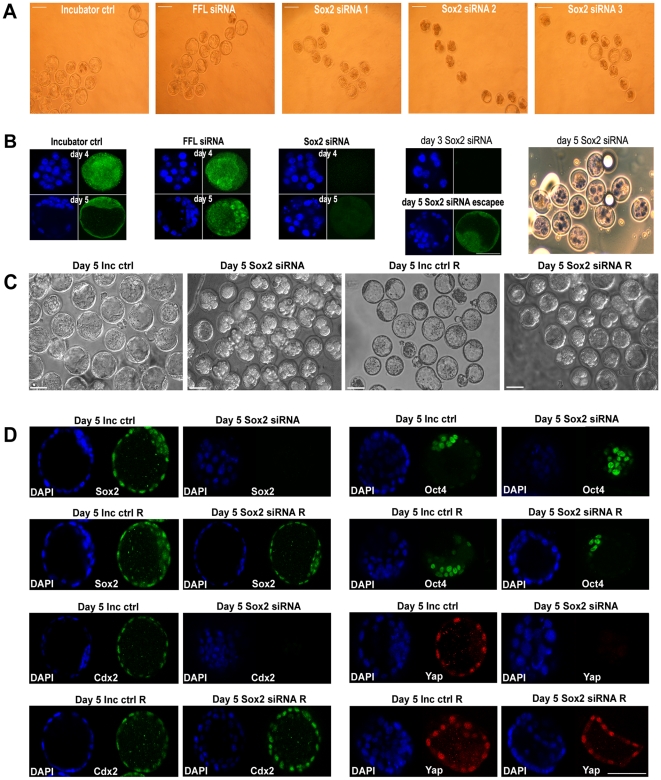
Sox2 expression in *Sox2-*siRNA and control *MF1*x*CD1* embryos. (**A**) Phase contrast images of day 5 incubator-control, *FFL*-siRNA, and *Sox2*-duplex-1, *-*2 and -3 siRNA embryos. While control embryos formed blastocysts, most *Sox2*-siRNA embryos did not. Bars: 100 µm. (**B**) Control and *Sox2*-duplex-2-siRNA embryos stained for Sox2 (Abcam) on days 4 and 5: single optical sections from confocal Z-series, DAPI-stained nuclei (blue) and Sox2 (green); bar: 50 µm. Decreased Sox2 protein expression was observed in *Sox2* knock-down morulae on day 4, which persisted on day 5. Absence of Sox2 protein on day 3, as a result of the *Sox2*-siRNA knock-down on day 2, was also confirmed. A few blastocysts forming on day 5 within the *Sox2*-siRNA group (‘escapees’ from the siRNA effect) showed some Sox2-staining, although less than incubator-control blastocysts. The lethality of the *Sox2*-siRNA phenotype on day 5 was assessed by Trypan Blue up-take. Presence of non-viable (blue) cells was apparent in 10 out of the 12 day 5 *Sox2*-siRNA embryos examined. (**C**) Rescue phenotype in *MF1*x*CD1* embryos: *Sox2*-siRNA and incubator-control embryos were cultured with and without cell-permeant Sox2 protein, and embryo development was assessed up to day 5. Phase contrast images of blastocysts from untreated day 5 incubator-control embryos (Day 5 Inc Ctrl); Lack of blastocyst formation in untreated day 5 *Sox2*-siRNA (duplex-2) embryos (Day 5 Sox2 siRNA); Blastocysts and some arrested embryos in day 5 control embryos treated with cell-permeant Sox2 protein (Day 5 Inc Ctrl R); Rescue phenotype on day 5 with blastocyst formation as well as some arrested embryos after treatment of *Sox2*-siRNA (duplex-2) embryos with cell-permeant Sox2 protein (Day 5 Sox2 siRNA R). Bars: 100 µm. (**D**) Comparison of D5 *Sox2* siRNA embryos with untreated incubator control embryos (D5 Inc ctrl), as well as with rescued D5 *Sox2* siRNA R embryos and D5 Inc ctrl R embryos for Sox2 (R&D), Oct4 (BD Biosciences), Cdx2 (Biogenex) and Yap (Cell Signaling) proteins. The images are single optical sections from confocal Z-series. Untreated D5 Inc ctrl blastocysts expressed Sox2 in ICM and TE nuclei, Oct4 in ICM nuclei, Cdx2 and Yap in TE nuclei; D5 *Sox2* siRNA arrested morulae did not demonstrate Sox2, Cdx2 and Yap staining, but expressed Oct4 in inner cell nuclei; D5 Inc ctrl R blastocysts showed similar expression patterns with D5 Inc ctrl blastocysts for all 4 markers; rescued D5 *Sox2* siRNA blastocysts expressed Sox2 in ICM and TE nuclei, Oct4 in ICM nuclei, Cdx2 and Yap in TE nuclei, indicating reversal of the *Sox2* siRNA phenotype. At least 10 embryos were stained for each antigen and representative embryos are shown. Similar results were obtained for both *Sox2* siRNA duplexes. Blue indicates DAPI stained nuclei and green/red staining for the protein of interest. In all cases immunological controls were negative (Figure S6). Bars: 100 µm.

*Sox2-*siRNA duplexes were electroporated separately; embryos were cultured and scored daily until day 4 and 5 of development (plug  =  day 1), when control embryos were at the morula (16–32 cells) and blastocyst stage respectively. No difference in development between groups was detected until day 4. The effects of the three *Sox2*-siRNA duplexes are presented in Figure 2. On day 4, the majority of embryos of all groups formed morulae (Figure 2A, 2C, 2E) at percentages from 60% to 73.5%. All *Sox2*-siRNA duplexes were effective in perturbing development; by day 5, *Sox2*-duplex-1 had led to developmental arrest of 24% of embryos at the 4-8-cell stage and 51.7% at the morula stage. *Sox2*-duplex-2 resulted in arrest mainly at the morula stage (71.5%). *Sox2*-duplex-3 caused 69% arrest at the morula stage. Morulae were compact and showed similar organization to control groups. Only 18.7% of the *Sox2*-duplexes -1 and -2, and 21% of the *Sox2*-duplex-3-siRNA embryos formed blastocysts, compared to 76.2% to 83% for incubator, 61.8% to 62.5% for *Galanin* and 55.4% to 56.5% for *FFL* controls respectively (Figures 2B, 2D, 2F and 3A). Thus absence of Sox2 prevented mouse preimplantation embryos developing beyond the morula stage. *Sox2*-duplex-1 appeared to have a more severe effect than duplexes -2 and -3, since a greater proportion of the embryos remained at the 4-8-cell stage rather than forming compacted morulae.

In order to rule out that the effects observed were not specific to the cross used (*MF1*x*CD1*), we also examined the effect of *Sox2*-duplex-1-siRNA on *MF1*x*MF1* embryos. As observed in the *MF1*x*CD1* embryos, after *Sox2*-siRNA knock-down >90% of the embryos underwent compaction but arrested at the morula stage (Figure S2).

To determine whether developmental arrest resulted from loss of Sox2 protein, we assessed protein expression by immunofluorescence with three different Sox2 antibodies (Figures 3B, 3D and S3) in *Sox2*-siRNA treated embryos at days 4 and 5 (*MF1*x*CD1*). Control groups (incubator-control, *Galanin*- and *FFL*-siRNA embryos) showed strong staining for Sox2 (Figure 3B). On both days, all embryos in the *Sox2*-siRNA group that remained at the morula stage were negative for Sox2 or showed extremely faint Sox2 staining. However, embryos in this group which reached the blastocyst stage on day 5, invariably showed Sox2 staining (Figure 3B), although generally decreased compared to control blastocysts. Therefore some of the *Sox2*-siRNA treated embryos may have escaped the RNAi effect and maintained Sox2 levels sufficient for blastocyst development. Compared to controls, there was a general delay in time of formation of the few blastocysts within the *Sox2*-siRNA embryos. Assessing levels of fluorescence after Sox2 staining, and dividing the number of embryos negative for Sox2 by the total number of embryos electroporated and examined per experiment, the knock-down efficiency after siRNA was found to be 71%, whereas 29% escaped the RNAi effect. Lethality of the *Sox2*-siRNA phenotype on day 5 was confirmed by Trypan-Blue uptake (Figure 3B). The majority (83%) of the knock-down embryos assessed for viability on day 5, contained a significant number of necrotic (blue-stained) cells. Therefore further follow-up of the *Sox2*-siRNA knock-down phenotype (e.g. embryo transfer) was not possible.

**Figure 4:**
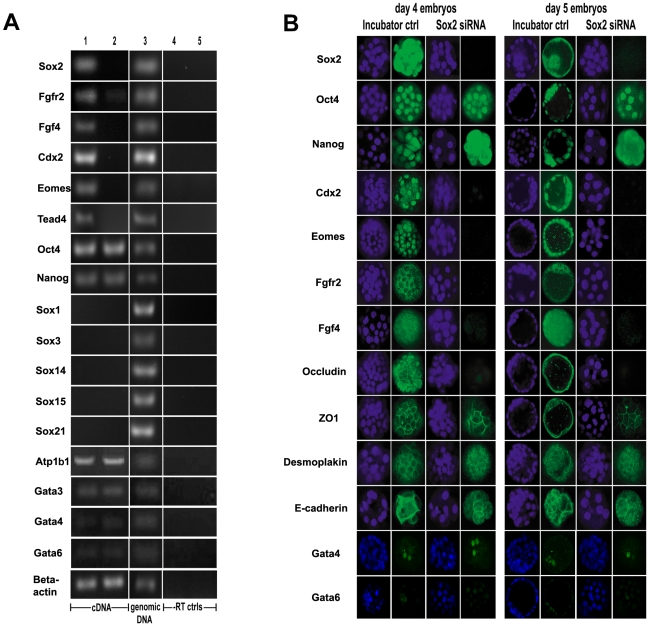
Assessment of ICM and TE markers in *Sox2*-siRNA and control *MF1*x*CD1* embryos. (**A**) RT-PCR for *Sox2*, *Fgfr2*, *Fgf4*, *Cdx2*, *Eomes*, *Tead4*, *Oct4*, *Nanog*, *Sox1*, *Sox3*, *Sox14*, *Sox15*, *Sox21* and *beta-actin* (40 cycles) on day 4: incubator-control embryos (lane 1); *Sox2*-duplex-2-siRNA embryos (lane 2); genomic DNA (lane 3); incubator-control morulae –RT (lane 4); *Sox2-*duplex-2-siRNA morulae –RT (lane 5). In the absence of *Sox2* transcript after siRNA, a clear reduction of *Fgfr2*, *Ffg4*, *Cdx2, Eomes* and *Tead4* transcripts in *Sox2*-siRNA embryos was observed. *Oct4* and *Nanog* transcripts were unaffected in *Sox2* knock-down morulae compared to incubator-control morulae. To ensure absence of signal did not reflect low detectable message, we performed PCR at 40 cycles. PCR for all transcripts was determined at 25, 30 and 35 cycles with results similar to those presented, but with weaker band intensity where present. *Sox1*, *Sox3*, *Sox14*, *Sox15* and *Sox21* transcripts were not detected in incubator-control morulae or *Sox2* knock-down morulae. *Atp1b1* (ATPase, Na+/K+ transporting, beta 1 polypeptide), *Gata3*, *Gata4* and *Gata6* transcripts were detected in comparable levels in *Sox2* knock-down morulae compared to incubator-control morulae. *Beta-actin* transcripts were detected in both *Sox2-*siRNA and incubator-control embryos. This figure illustrates *Sox2*-duplex-2-siRNA embryo transcripts, whereas similar patterns of transcript expression for *Sox2*-siRNA duplexes -1 and -3 are shown in Figure S4. (**B**) Comparison of *Sox2* knock-down embryos (as assessed by absence of fluorescence after Sox2 staining) with incubator (untreated) control embryos for ICM and TE markers. Immunofluorescence for Sox2 (Abcam), Oct4, Nanog, Cdx2 (rabbit polyclonal from Jane Collins), Eomes, Fgfr2, Fgf4, Occludin, ZO1, Desmoplakin, E-cadherin, Gata4 and Gata6 to compare protein expression between incubator control and *Sox2*-siRNA embryos at day 4 and 5 of preimplantation development. The images are single optical sections from confocal Z-series. *Sox2*-duplex-1-siRNA embryos stained for Sox2 are shown in this Figure. In all cases, after knock-down with each of the three duplexes, *Sox2* siRNA embryos were negative for Sox2. For assessment of the other markers, embryos from all three knock-down construct groups were pooled. A large reduction in Sox2, Cdx2, Eomes, Fgfr2, Fgf4 and Occludin protein was observed, whereas Oct4, Nanog, ZO1, Desmoplakin, E-cadherin, Gata4 and Gata6 remained unaffected. However, Nanog staining appeared more cytoplasmic in *Sox2* knock-down embryos. The selected image for the day 4 incubator control embryo stained for Cdx2, is a plane through the top of the embryo and the positively stained Cdx2 cell in the center is located in the outer layer of the embryo. The staining patterns for Sox2 and Cdx2 were confirmed with different Sox2 (Chemicon) and Cdx2 (Biogenex) antibodies (Figure S3). At least 10 embryos were stained for each antigen and representative embryos are shown. Blue indicates DAPI stained nuclei and green staining for the protein of interest. In all cases immunological controls were negative (Figure S6). Where antibodies were compatible, embryos were double-stained for two markers. In all cases the anti-Sox2 staining for *Sox2*-siRNA embryos was negative. Bar: 50 µm.

The *Sox2* knock-down was also confirmed by RT-PCR in pools of 30 embryos. A loss of *Sox2* transcript in day 4 *Sox2*-siRNA morulae (Figures 4A and S4) was observed compared to incubator-control (untreated) morulae, in which *Sox2* transcripts were always detected. To determine whether the developmental arrest of *Sox2* RNAi treated embryos was associated with loss of other genes (some of them *Sox2* targets), we examined transcript expression of *Fgf4*, *Fgfr2*, *Oct4*, *Nanog*, *Cdx2*, *Eomes* and *Tead4* (Figures 4A and S4). In embryos where *Sox2* transcript was undetectable, *Fgfr2* transcripts were barely visible, *Fgf4*, *Cdx2*, *Eomes* and *Tead4* transcripts were untraceable after amplification of cDNA to 40 cycles, whereas *Oct4* and *Nanog* transcripts remained unaffected (Figures 4A and S4). Comparable transcript levels for *Atp1b1* (ATPase, Na+/K+ transporting, beta 1 polypeptide), *Gata3*, *Gata4* and *Gata6* were detected in *Sox2* knock-down morulae compared to incubator-control morulae (Figure 4A). This implies that loss of *Sox2* led to down-regulation of *Fgf4*, *Fgfr2*, *Cdx2*, *Eomes* and *Tead4* transcripts, but did not affect *Oct4*, *Nanog*, *Atp1b1, Gata3*, *Gata4* and *Gata6* transcripts in the morula. Similar levels of *βeta-actin* transcripts (control) were detected in *Sox2*-siRNA and incubator-control embryos.

Due to the similarity of sequence in the HMG domain (which *Sox2*-duplex-1 targeted), between *Sox2* and *Sox1*, *Sox3*, *Sox14*, *Sox15* and *Sox21*, the presence of the latter transcripts was also investigated to verify the specificity of the *Sox2* knock-down. Transcripts for none of these genes were detected at the morula stage in control or knock-down embryos after siRNA for each of the 3 duplexes (Figures 4A and S4). To confirm that the *Sox2* knock-down phenotype was due to specific depletion of Sox2, we performed rescue experiments using recombinant cell-permeant Sox2 protein fused with a TAT protein transduction domain to allow intracellular delivery, which has been previously shown to be highly efficient in compensating for loss of RNAi-induced knock-down of *Sox2* in ES cells [52].

The cell-permeant Sox2 protein was added 24 h after RNAi in the *Sox2* siRNA group as well as incubator control embryos and it was renewed every 24 h as reported in Materials and Methods. Up-take of the Sox2-TAT protein was monitored by immunofluorescence 6 h, 12 h and 18 h after its first addition. Gradual increase in Sox2 protein expression and signs of possible protein uptake through an endocytic pathway were detected, as demonstrated by the patchy Sox2 expression (Figure S5). A significant rescue phenotype was observed among the *Sox2* siRNA embryos by day 5 of development (Figures 2G, 3C and 3D). While 43–47.7% of the *Sox2* siRNA (duplexes 2 and 3) embryos arrested at morula stages with only 18.7–21% forming blastocysts, their equivalent *Sox2* siRNA R (rescue groups) formed blastocysts at percentages from 59.7% to 62.6% (Figures 2G, 3C), indicating substantial phenotype reversal upon addition of the Sox2 protein in the *Sox2* siRNA groups. At the same time, 72.75% of the untreated incubator control embryos formed blastocysts, whereas 50.9% of the embryos treated with the cell-permeant Sox2 protein (Inc ctrl R) reached the blastocyst stage (Figures 2G, 3C). A significant proportion of the remaining incubator control Sox2-treated embryos appeared perturbed at earlier stages, indicating some detrimental effect of higher than normal levels of Sox2 in these embryos.

### Loss of Sox2 does not affect Oct4 expression, influences Nanog localization but not expression, but causes downregulation of trophectoderm associated proteins

In order to investigate the *Sox2*-siRNA phenotype, we stained embryos for a number of ICM and TE markers, expressed at the morula and blastocyst stage (Figure 4B). Both day 4 and day 5 embryos were assessed after *Sox2*-siRNA for Sox2 protein expression, which was shown to be almost or completely absent. When *Sox2*-siRNA embryos were compared with incubator-control embryos, reduction in a number of TE proteins was observed. Yap, Cdx2, Eomes and Occludin were present in control embryos but could not be detected or were weakly detected in *Sox2*-siRNA embryos (Figures 3D and 4B). Fgfr2 and Fgf4 proteins were also decreased (Figure 4B), coinciding with the absence of transcripts for these and other TE markers (Tead4, Cdx2, Eomes) in *Sox2*-siRNA embryos (Figures 4A and S4). However, the early trophoblast stem cell marker Gata3, as well as Oct4, Nanog, ZO1, Desmoplakin, E-cadherin, Gata4 and Gata6 protein expression remained unaffected (Figures 3D and 4B) by *Sox2* knock-down. Although Nanog protein was expressed in day 4 and day 5 in *Sox2*-siRNA embryos, it did not appear to be located in the nuclei. In the absence of *Sox2* transcripts in *Sox2*-siRNA embryos, *Oct4* and *Nanog* transcripts remained unaffected (Figures 4A and S4). The rabbit polyclonal Cdx2 antibody gave more prominent nuclear staining than the mouse monoclonal Cdx2, however both stained TE nuclei and cytoplasm. Immunological controls for all antibodies were negative (Figure S6).

After reversal of *Sox2* knock-down phenotype, day 5 *Sox2*-siRNA R blastocysts expressed Sox2 in ICM and TE nuclei, Oct4 in ICM nuclei and Yap and Cdx2 in TE nuclei, resembling the day 5 incubator control and control R blastocysts (Figures 3B, 3D, 4B). The arrested day 5 *Sox2*-siRNA morulae did not demonstrate any Sox2, Yap or Cdx2 staining but did express Oct4 in inner cell nuclei (Figure 3D), resembling the originally observed *Sox2* knock-down phenotype (Figures 3B, 4B).

### Sox2 transient knock-down induces apoptosis at day 5 of development

**Figure 5:**
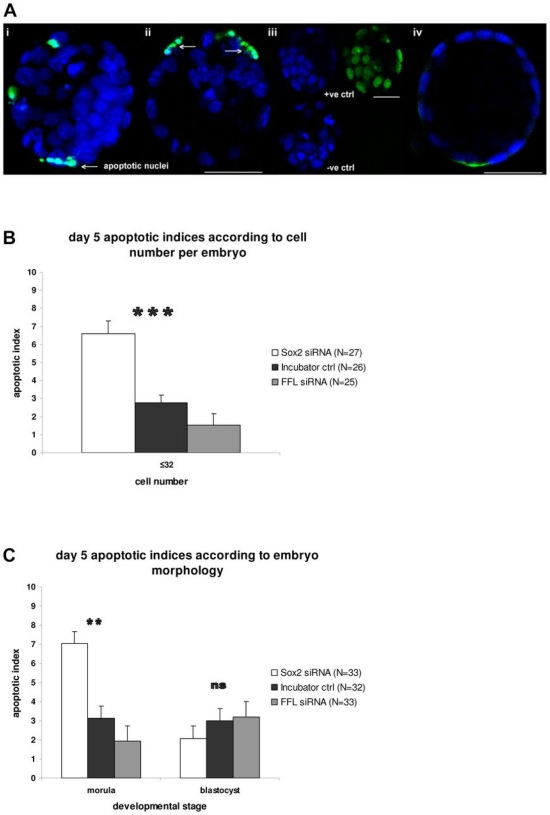
Assessment of apoptosis in embryos using the TUNEL assay. (**A**) Fluorescence analysis for apoptotic cells; the images are single optical sections from confocal Z-series. The fragmented nuclei (green fluorescence) are apoptotic (white arrows). i. Day 5 incubator-control embryo; ii. Day 5 *Sox2* knock-down embryo; iii. Controls for the TUNEL reaction (positive control top; negative control bottom; blue DAPI); iv. Day 5 *FFL*-siRNA embryo. Bars: 50 µm. (**B**) Mean apoptotic indices in embryos with ≤32 cells on day 5. *Sox2-*siRNA embryos (white, N = 27) were compared with incubator-control embryos (black, N = 26) and *FFL*-siRNA embryos (grey, N = 25). Independent samples T-test revealed significant difference (p<0.0001) between apoptotic indices of *Sox2*-siRNA and incubator-control as well as *FFL*-siRNA embryos with ≤32 cells. (**C**) Mean apoptotic indices for day 5 mouse embryos. *Sox2-*siRNA embryos (white, N = 33) were compared with incubator-control embryos (black, N = 32) and *FFL*-siRNA embryos (grey, N = 33). Independent samples T-test revealed a significant difference (p = 0.003) between apoptotic indices of *Sox2-*siRNA and incubator-control or *FFL*-siRNA morulae. There was no statistically significant difference (p = 0.2) between apoptotic indices of *Sox2-*siRNA escapee blastocysts, incubator-control and *FFL*-siRNA blastocysts. Embryos from all three *Sox2* knock-down groups were pooled.

**Figure 6:**
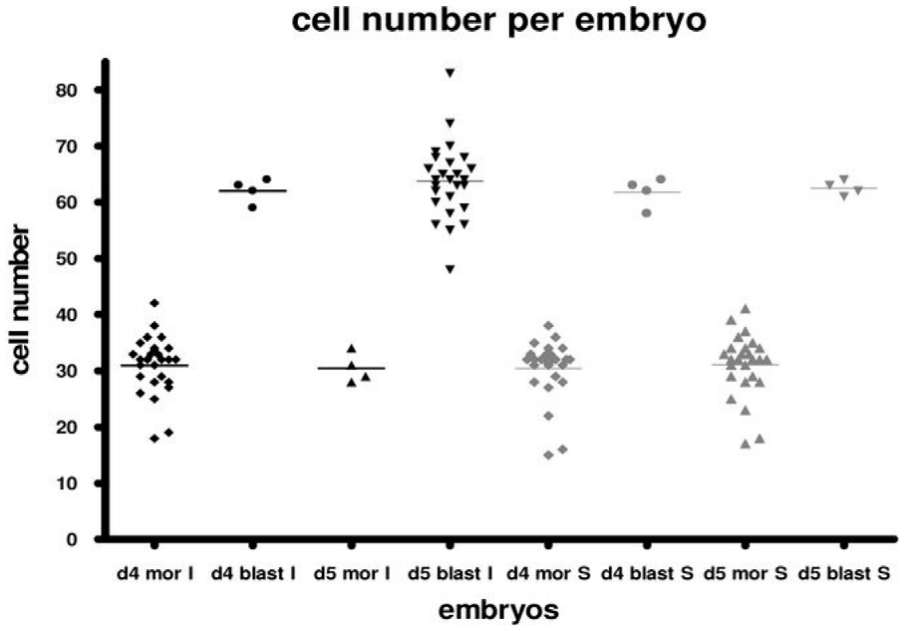
Assessment of cell number per embryo. Cell number per embryo in *Sox2-*siRNA (grey) and incubator-control morulae and blastocysts (black) at days 4 and 5 of preimplantation development. The *Sox2*-siRNA embryos were confirmed to be *Sox2* knock-down embryos, as assessed by absence of fluorescence after Sox2 immunostaining. Day 4 *Sox2-*siRNA morulae (d4 mor S, N = 26) were compared with day 4 incubator-control morulae (d4 mor I, N = 26) and day 4 *Sox2*-siRNA blastocysts (d4 blast S, N = 4) were compared with day 4 incubator-control blastocysts (d4 blast I, N = 4). Day 5 *Sox2*-siRNA morulae (d5 mor S, N = 26) were compared with day 5 incubator-control morulae (d5 mor I, N = 4) and day 5 *Sox2*-siRNA blastocysts (d5 blast S, N = 4) were compared with day 5 incubator-control blastocysts (d5 blast I, N = 26). Chi-square tests revealed that there was no statistically significant difference between these groups. Thus arrested *Sox2*-siRNA morulae remain at the cell number for the morula stage when control embryos have divided further and formed blastocysts. Embryos from all three *Sox2* knock-down groups were pooled.

*Sox2*-siRNA, *FFL*-siRNA and incubator-control embryos were assessed for apoptosis at day 4 and 5 of development. While no apoptotic cells were identified at day 4 in either the control groups or the *Sox2*-siRNA groups, lack of Sox2 appeared to induce increased apoptosis in day 5 *Sox2*-siRNA embryos (Figure 5A), as indicated by an increased apoptotic index both in embryos with ≤32 cells (Figure 5B) and in arrested morulae (Figure 5C). The relative distribution of apoptosis between inner and outer cells (ICM and TE, respectively, where blastocysts had formed) in siRNA and control groups was the same (30% inner cells, 70% outer cells), thus most of the apoptosis appeared to be in the outer cells in all groups. Furthermore, *Sox2* knock-down did not affect total cell numbers when considering both morulae and blastocysts at day 4 or 5 (Figure 6). Nevertheless, arrested morulae in the *Sox2* knock-down group had lower cell numbers than control blastocysts. To conclude, more apoptotic cells were observed in *Sox2* knock-down embryos compared to control embryos. However when compared by morphology, morulae and blastocysts (incubator-control versus *Sox2*-siRNA ‘escapee’ blastocysts) of both groups contained the same number of cells.

## Discussion

### Sox2 expression in mouse preimplantation development

We found that Sox2 is expressed throughout preimplantation mouse development with cytoplasmic or nuclear Sox2 protein detected from the unfertilized oocyte to the blastocyst stage. This finding is partially in agreement with Avilion et al. [4] who showed that Sox2 protein was present in the cytoplasm of the fertilized oocyte, but its translocation into nuclei occurred at the 2-cell stage. Moreover, in our study, *Sox2* transcripts of maternal origin were detected in oocytes and Sox2 protein was found in both nucleus and cytoplasm of the oocyte. Some 4-cell embryos had mainly cytoplasmic staining while others mainly nuclear; indeed some had blastomeres in both categories. This may relate to the increasing zygotic transcription at the 4-cell stage and suggests that Sox2 nuclear translocation may be initiated a bit later, at the 4-cell stage, in the strain of mice we examined compared to that used by Avilion et al. In later cleavage-stage embryos nuclear staining was invariably observed. The strong expression of Sox2 in TE and ICM at the blastocyst stage is generally in agreement with Avilion et al. [4]. They showed that in early blastocysts Sox2 was uniquely cytoplasmic in TE cells, whereas we observed nuclear and cytoplasmic expression of Sox2 in some TE cells. They reported that Sox2 was mostly nuclear in ICM and our observations are in agreement.

### Role of Sox2 in mouse preimplantation development

The aim of our study was to define the effect of knocking down *Sox2* in developing preimplantation mouse embryos, in order to uncover early functions in maintaining developmental potential of early mouse embryos and marking the pluripotent lineage [53]. After knock-out, in the absence of zygotic *Sox2*, the epiblast was not maintained but rather differentiated into diverse cell types [4]. The authors suggested that maternal Sox2 protein derived from the oocyte may carry the embryo through preimplantation development in the absence of oocyte *Sox2* mRNA. However, in our study *Sox2* transcripts were detected in oocytes and siRNA for *Sox2* led to developmental arrest at the morula stage, suggesting a significant contribution of maternal *Sox2,* and that Sox2 protein and transcripts are crucial in the mouse preimplantation embryo for an additional earlier cell function than previously described. When *Sox2*-siRNA is applied early in preimplantation development, Sox2 protein levels are barely detectable or absent 3 days later, confirming the efficacy of siRNA as a technique for examining preimplantation protein function [54]–[56]. After knocking down *Sox2* in 2-cell mouse embryos with three different siRNA duplexes, most morulae had very low levels of Sox2 and failed to develop to blastocysts, while this effect was not seen in control groups. The slightly smaller numbers of blastocysts forming after *Galanin*- and *FFL*-siRNA compared with incubator-control embryos suggests that the technique of siRNA causes some stress to embryos, but without statistically significant effect on development. This is further supported by the viability and normal post-implantation development following Galanin siRNA by electroporation in 2-cell stage mouse embryos and subsequent embryo-transfer at the blastocyst-stage to pseudopregnant mice (J. Razavi, D. Brison and S. Kimber, unpublished). The difference of severity of the *Sox2* knock-down outcome when using the two different crosses (*MF1*x*MF1*, *MF1*x*CD1*) may be explained in the light of different genetic background and possibly dosage or polymorphisms of interacting genes of the mouse strains [57], [58].

*Sox2*-duplex-1 had a stronger impact on developing mouse embryos than duplexes -2 and -3, causing a proportion of them to arrest earlier, namely at the 4-8-cell stage. However, the *Sox2*-duplex-1 was designed to target within the conserved region of the HMG box, and might affect additional genes. We have controlled for this by using two siRNA sequences designed outside the HMG box and targeting *Sox2* alone. In this case, for most of the *Sox2* knock-down embryos developmental arrest occurred at the transition from the morula to the blastocyst stage, with *Sox2*-siRNA morulae failing to cavitate or undergo cell division beyond the 32-cell stage. However, a small number of *Sox2*-siRNA embryos ‘escaped’ the RNAi effect. These developed to blastocysts and showed moderate levels of Sox2 protein expression, though generally lower than control blastocysts. This indicates that in some embryos knock-down is incomplete, allowing sufficient Sox2 protein to accumulate by the late morula stage to permit development to the blastocyst stage. Thus a threshold level of Sox2 might be required for the developmental transition from a compacted morula to a fully differentiated blastocyst. Furthermore, *Sox2*-siRNA ‘escapee’ embryos were generally delayed in forming blastocysts compared to incubator-control embryos, whereas arrested *Sox2*-siRNA morulae did not form TE or a cavity when left in culture. This observation was further supported by confirming the lethality of the *Sox2*-siRNA phenotype on day 5, with the majority of the embryos becoming necrotic, as indicated by incorporation of Trypan-Blue.

After rescue experiments, using Sox2 protein fused with a TAT protein transduction domain [52], the *Sox2*-siRNA phenotype was reversed and blastocysts were formed. This confirms that *Sox2* depletion was indeed the cause of the siRNA induced phenotype. The same cell-permeant Sox2 protein has been successfully used to rescue the stem cell phenotype observed in mES cells after *Sox2*-siRNA knock-down [52]. Indeed, a series of recent publications demonstrate that TAT-mediated protein transduction is an efficient strategy to deliver biologically active proteins such as transcription factors into a variety of cells [59]–[61], also reviewed in [62].

### Loss of Sox2 does not affect Oct4 and Nanog expression, but causes downregulation of trophectoderm associated proteins

Loss of *Tead4* (a molecule recently shown to be essential for TE formation in preimplantation mouse embryos [39]), Cdx2 and Eomesodermin, as well as the Tead4 coactivator protein Yap, (which localizes in nuclei of outer TE cells before Cdx2 does [39]), occurred after *Sox2* knock-down in mouse embryos. Moreover, regular expression of all these genes after Sox2-mediated rescue of blastocysts, indicates that Sox2 may regulate genes that control differentiation of the outer cells of the morula to form TE. This is supported by the reduction in cell surface expression of Occludin, a component of tight junctions [63]. However since ZO1, Desmoplakin and E-cadherin protein expression, as well as Na+/K+-ATPase β-subunit (*Atp1b1*) transcript expression were not affected, the initiation of epithelia formation appears to be intact in *Sox2* knock-down embryos. The absence of Fgfr2 in *Sox2*-siRNA embryos also implies malfunction of the outer cells of the embryo, whereas the reduction of *Fgf4*, a direct target gene of the Sox2-Oct4 synergy, would reflect malfunction of the ICM [64]. In this respect, the identification of *Sox2* transcripts as the earliest marker of inner cells of the embryo should be noted [19]. *Tead4* −/− embryos [39] arrested at the morula stage, did not form blastocyst cavities and downregulated TE associated markers Cdx2, Eomes and Fgfr2. Similarly, in *Tead4* −/− arrested morulae, E-cadherin was not affected [39]. The striking similarity between the *Sox2* RNAi embryo phenotype and that of the *Tead4*-null embryos, suggests a potential link. The early trophoblast marker Gata3 is expressed along with Cdx2 during preimplantation development and also maintained by Tead4 although expressed independently of Cdx2 [40]. Gata3 also regulates trophectoderm, but it did not seem to be affected by the absence of Sox2 in our study, implying the existence of still poorly defined transcription factor circuitries affecting cell fate decisions [65].

*Sox2* knock-down did not affect total cell numbers up to the morula stage (days 3–4). However, whilst control embryos cultured up to day 5 (approximately 72 h post 2-cell stage) reached roughly 64 cells and formed blastocysts, *Sox2* knock-down embryos did not progress beyond 30 cells. In *Tead4* −/− embryos cell division occurred on schedule up to 60 h post 2-cell stage, reaching 32 cells and a morula-like morphology, which was maintained, as embryos did not form clear blastocyst cavities. The authors stated that cell proliferation in the *Tead4* −/− embryos was not arrested or significantly delayed [39] although they did not examine cell numbers later than 60 h post 2-cell. In our study, *Sox2* RNAi embryos also failed to form blastocyst cavities and arrested at the morula stage. Sox2 may facilitate the maintenance of cell division in the embryo of Oct4 +ve cells, since embryos did not continue beyond the 30-cell stage when it was depleted. Sox2 could potentially regulate TE formation in the preimplantation embryo at some point upstream of Tead4, since *Tead4* expression is affected in the absence of *Sox2* (this study) but *Sox2* is not affected in the absence of *Tead4* [39]. The lack of blastocyst cavity formation observed in our *Sox2* RNAi embryos, as well as in the *Tead4* −/− embryos [39], suggests a more severe phenotype than in *Cdx2* −/− embryos, which gradually cavitate despite the absence of Cdx2 [38]. Furthermore, whilst *Sox2* RNAi and *Tead4* −/− embryos downregulate Cdx2, Eomes and Fgfr2, *Cdx2* −/− embryos express Eomes (though weakly) and Fgfr2 [38]. In addition, the absence of the Tead4 co-activator and upstream Cdx2 regulator protein, Yap, in day 5 *Sox2*-siRNA embryos, but its presence in the rescued *Sox2*-siRNA blastocysts, make Sox2 a likely early regulator in the sequence of events leading to TE formation in the preimplantation mouse embryo. The observation that rescued *Sox2*-siRNA blastocysts express protein for Sox2 in ICM and some TE nuclei, Oct4 in ICM nuclei, as well as Cdx2 and Yap in TE nuclei, and now form blastocysts, is also consistent with Sox2 involvement in regulating the trophectoderm associated markers Yap and Cdx2, whether directly or indirectly.

In our study, the pluripotency marker proteins Oct4 and Nanog remained unchanged despite the lack of Sox2. This is surprising taking into account their interconnected regulation in ES cells. A similar maintenance of pluripotency markers was also observed in the absence of Tead4 [39]. Enhancers of a number of pluripotency associated genes contain a *Sox*-*Oct* element, which both Sox2 and Oct4 bind in a combinatorial interaction [24]. The complex formed regulates several genes (among them *Oct4* and *Sox2* themselves, as well as *Nanog*) that have been identified as essential for the formation of the ICM during mouse preimplantation development and for self-renewal of pluripotent ES cells [4], [20], [25], [26]. The retention of Oct4 and Nanog expression, despite the depletion of Sox2 in this study, should be viewed in the light of their own auto-regulation which is likely to allow their transcription and translation even in the absence of Sox2. Alternatively, the well described trio-motif-network of Sox2-Oct4-Nanog might not function at the morula stage, so that Oct4 and Nanog can persist in the absence of Sox2. This network may only come into force at the blastocyst stage, when formation of the ICM leading to the epiblast occurs. However, Nanog expression appeared more cytoplasmic in *Sox2*-siRNA embryos compared to controls. Since Nanog is first expressed at the late morula stage [25], [26], it is likely that after *Sox2* knock-down, embryos arrest at about the time Nanog is first transcribed/translated and hence staining may represent newly synthesised cytoplamic protein, not yet translocated into the nucleus [25], [26]. Since the *Sox2* knock-down embryos are delayed compared to controls, this might explain why control morulae already show a strong nuclear Nanog signal.

Fgf4 is expressed at the blastocyst stage [45], [66] and regulates the 5^th^ cell cycle in the mouse embryo [67]. Thus loss of Fgf4 could account for the predominance of morulae with less than 32 cells after *Sox2*-siRNA. Later Fgf4, originating from the ICM and epiblast, acts upon TE cells in a paracrine manner [37]. The *Fgf4* gene has both core HMG and POU regulatory elements that are mutually dependent, and an additional independent *Sox2*-binding HMG regulatory element [68]–[70]. In *Sox2*-siRNA embryos both Fgf4 and Fgfr2 were downregulated, and there were almost undetectable levels of the TE markers Cdx2 and Eomes. FGF signalling is essential for the regulation of Cdx1, Cdx2 and Cdx4 during gastrulation in *Xenopus laevis* [71]. Therefore it is possible that Fgf4/Fgfr2 signalling induces Cdx2 and Eomes, thereby facilitating TE formation. On the other hand Sox2 may be required in early cells expressing Cdx2 or for its maintenance. In its absence, Cdx2 is not expressed, with concurrent lack of Eomes and Fgfr2 expression. It has previously been shown that Eomes is downstream of Cdx2 in TE differentiation [38], [49]. Furthermore, the parallel expression of Fgfr2 and Cdx2 in outer cells of the preimplantation embryo [48], and the established role of FGF signalling at the 5^th^ cell cycle when overt TE is generated [67], could imply a concurrent requirement for FGF signalling with Cdx2 expression during TE development. The association of Sox2 and Cdx2 genes has also been shown in a different system, that of gastric cancer where Sox2 and Cdx1/Cdx2 are inversely related [72], [73]. Interestingly, the extra-embryonic endoderm markers Gata4 and Gata6 were not affected by *Sox2*-siRNA, showing similar patterns of expression as previously reported [74], [75]. Our finding therefore reinforces previous observation that Sox2 does not seem to exert an effect on extra-embryonic endoderm [4].

Recent findings in mES cells show an inverse relationship between Sox2 and TE associated markers, as ES cells can differentiate into trophectoderm in the absence of Sox2 [15], [76, Keramari, Ward and Kimber unpublished]. However ES cells are derived from the ICM, generally from blastocysts in implantation delay, and are a completely different cell population to the cleavage and morula stage embryo investigated in our study. Murine ES cells do not, unless experimentally forced, differentiate to TE [77], while the outer cells of the morula spontaneously differentiate to TE in utero or in simple media in vitro [78]. Furthermore, the cross-talk between ICM and TE that takes place in the embryo is different from the signalling between ES cells in culture. It has also been shown that Sox proteins can act either as activators or repressors in different cellular contexts [79], and that critical thresholds of Sox2 are associated with different phenotypes in early cell lineage commitment events in neural and foregut endoderm differentiation [80], [81]. This could explain the observation that Sox2 seems to positively regulate Cdx2 and Eomes in the morula, but to repress both genes in ES cells [15], [76, Keramari et al. unpublished]. For example, it has been reported that a single transcription factor, namely Sall4, regulates two distinctively different stem cell populations within the blastocyst, ES cells arising from the ICM, and XEN stem cells originating from the extra-embryonic endoderm [82]. Sall4 is interconnected with the Sox2, Oct4 and Nanog auto-regulatory circuit to preserve pluripotency of ES cells, but at the same time induces genes critical for extra-embryonic endoderm formation like *Gata4*, *Gata6*, *Sox7* and *Sox17* [82]. Furthermore, it was recently shown that the transcription coactivator Yap, previously thought to regulate only trophectoderm in the mouse embryo [39], also seems to exert a role in regulation of self-renewal and differentiation in mouse ES cells [83]. This illustrates the common finding that transcriptional regulators of lineage segregation events can exert distinctly different functions within closely associated cell populations. In addition, it has been demonstrated that Nanog, previously thought to be a core transcription factor of the network that regulates pluripotency, though essential for ICM and germ cell formation, is dispensable for murine ES-cell self-renewal [84].

Our results lead us to conclude that Sox2 plays two distinctively different roles at two different early developmental stages, namely the morula and the ICM-derived ES cells and early epiblast [4], [15], [19, our data]. In addition, the association of Sox2 with Yap/Tead4/Cdx2/Eomes/Fgfr2 expression and TE formation in the early mouse embryo arising from our study suggests Sox2 may play a role in regulation of TE fate.

### Sox2 transient knock-down induces apoptosis at day 5 of development

*Sox2*-siRNA embryos showed higher levels of apoptosis compared to incubator-control and *FFL*-siRNA embryos at day 5 of development. Electroporation did not appear to cause increased apoptosis, since *FFL*-siRNA embryos exhibited similarly low levels of apoptosis to the incubator-control embryos. Therefore knock-down of *Sox2* seems to induce apoptosis, suggesting that Sox2 may regulate survival factors. Interestingly, in all groups, there were more apoptotic cells among outer (TE) cells than inner (ICM) cells. This may indicate a strain difference since most studies [85], [86] revealed higher apoptosis in the ICM than trophectoderm of mouse embryos cultured from the zygote to the blastocyst stage. Although many studies have shown equal levels of apoptosis between ICM and TE in human embryos, Hardy et al. [87] reported increased apoptosis in TE. Strumpf et al. [38] demonstrated that *Cdx2* disruption caused peri-implantation embryonic lethality due to lack of establishment of the TE. *Cdx2* mutant morulae initiated cavitation and cellular polarisation for TE commitment, but ultimately cells underwent apoptosis. The potential link between Sox2 and Cdx2, revealed by our study, concurs with the similar induction of apoptosis when either Sox2 or Cdx2 are eliminated.

In summary, after knocking down *Sox2* in 2-cell stage mouse embryos using siRNA, most embryos arrested at the morula stage and apoptosis was increased. While pluripotency markers Oct4 and Nanog remained unaffected, trophectoderm markers Tead4, Yap, Cdx2, Eomes and Fgfr2 were downregulated. The RNAi phenotype was rescued by introduction of Sox2 protein fused with a TAT protein transduction domain, leading to blastocyst formation and re-expression of Sox2, Yap and Cdx2. This indicates that Sox2 plays an important role in the preimplantation mouse embryo and its *earliest* essential function appears to involve establishment of the trophectoderm lineage either by directly or likely indirectly regulating Tead4 and Cdx2, or alternatively through Fgf4-Fgfr2 signalling. Future experiments would be essential in order to investigate further the mechanism by which Sox2 regulates these or other signalling pathways to promote trophectoderm induction in the preimplantation embryo. Furthermore, it has become apparent that *Sox2* maternal transcripts and protein are likely to play an important role in the earliest function of Sox2.

## Materials and Methods

### Ethics Statement

This study was approved by the Ethics Review Committee of the University of Manchester and the UK Home Office (Licence Numbers 40/1939 and 40/2669).

### Animals

Outbred female *MF1* mice (Harlan Olac Ltd, Bicester, UK, as used in our previous work) were crossed with male *CD1* mice with some preliminary data obtained from *MF1*x*MF1* crosses. Mice were kept under standard conditions (20–22°C, 40–60% humidity, 12 h light/dark cycle). Female mice were superovulated with 5 IU of pregnant mare's serum gonadotrophin (PMSG, Calbiochem) by intraperitoneal injection (0.1 ml). Ovulation was synchronised by a 5 IU intraperitoneal injection (0.1 ml) of human chorionic gonadotrophin (hCG, Intervet) 46–48 hours later. Female mice were paired with males overnight and monitored the following morning for the presence of a vaginal plug, which was designated day 1 of pregnancy.

On day 2 female mice were killed by cervical dislocation, oviducts were dissected and transferred into M2 medium (Sigma) with 4 mg/ml bovine serum albumin (BSA, ICN Biomedical) before flushing. Embryos were flushed using sterile M2/BSA and a 34-Gauge blunt-ended stainless steel needle (Coopers Needleworks) attached to a 1 ml syringe.

### Mouse embryo culture

Embryos were washed and cultured in a 30 µl drop of KSOM (EmbryoMax, Chemicon), under embryo-tested mineral oil (Sigma) at 37°C with 5% CO_2_ as described previously [58].

### Mouse ES cell neural differentiation protocol

*E14* mouse ES cells were cultured as described by Ying et al. [88], in order to be induced to differentiate towards neuroectodermal precursors. The medium was renewed every two days and the cells were cultured for 14 days. They were then fixed and immunostained as embryos, described below.

### RNA interference (RNAi)

Specific oligonucleotide RNAi duplexes (Eurogentec) to *Sox2* (NM_011443), *Galanin* (NM_010253) and *Firefly Luciferase* (*FFL*, D25416, *Photuris pennsylvanica luciferase*) were designed, using the Dharmacon short interference RNA (siRNA) oligo-designer-tool (http://design.dharmacon.com/default.aspx), taking into account 8 criteria for siRNA specificity and efficiency, as determined by Reynolds et al. [89]. Sequences were copied into the NCBI BLAST database to confirm matching with the sequences of interest. *Sox2*-duplex-1 was designed to target *Sox2* sequence within the HMG box, also found in other *Sox* genes; *Sox2*-duplexes -2 and -3 were designed to target *Sox2* sequences outside the conserved region of the HMG box (both homologous to *Sox2* only). Sense and antisense siRNA oligos used for electroporation are as follows:

*Sox2-*duplex-1 sense: 5′-GAUGCACAACUCGGAGAUCdTdT-3′

*Sox2-*duplex-1 antisense: 5′-GAUCUCCGAGUUGUGCAUCdTdT-3′

*Sox2*-duplex-2 sense: 5′-ACAGCUACGCGCACAUGAAdTdT-3′

*Sox2*-duplex-2 antisense: 5′-UUCAUGUGCGCGUAGCUGUdTdT-3′

*Sox2*-duplex-3 sense: 5′-GCACAUGAACGGCUGGAGCAAdTdT-3′

*Sox2*-duplex-3 antisense: 5′-UUGCUCCAGCCGUUCAUGUGCdTdT-3′

*Galanin-*duplex-1 sense: 5′-GCAACAUUGUCCGCACUAUdTdT-3′

*Galanin-*duplex-1 antisense: 5′-AUAGUGCGGACAAUGUUGCdTdT-3′

*Galanin*-duplex-2 sense: 5′-UAAUGGAGUUUCUCAGUUUdTdT-3′

*Galanin*-duplex-2 antisense: 5′-AAACUGAGAAACUCCAUUAdTdT-3′

*Galanin-*duplex-3 sense: 5′-AGAGGGAGUUACAACUGGAdTdT-3′

*Galanin-*duplex-3 antisense: 5′-UCCAGUUGUAACUCCCUCUdTdT-3′

*FFL* sense: 5′-CUCUUCAGGUUCUACGGGAdTdT-3′

*FFL* antisense: 5′-UCCCGUAGAACCUGAAGAGdTdT-3′

The 5′-end of each sense oligo was conjugated to rhodamine. 30 µl of each sense and antisense oligo from the stock solutions (100 µM) were mixed and 15 µl of annealing buffer (50 mM Tris/pH 7.5, 100 mM NaCl, Eurogentec) was added. Oligos were annealed by heating to 95°C for 2 minutes and then allowed to cool for 1 hour at 4°C. The annealed duplex was diluted 1∶20 in 4-(2-hydroxyethyl)-1 piperazine ethanesulfonic acid (HEPES)-buffered saline (HBS) supplemented with 4 mg/ml BSA to give a final concentration of 2 µM (individual *Sox2*-siRNA duplexes and *FFL*-siRNA duplex). Combination of the three *Galanin*-siRNA duplexes created a final concentration of 6 µM.

### Electroporation of mouse 2-cell embryos

Flushed 2-cell embryos were washed in HBS/BSA, transferred into the electroporation microchamber slides (VWR) in a minimal volume of medium and electroporated using an ECM®830 electroporator (VWR). Thirty embryos per group in 60 µl of each annealed siRNA (50 µM) were loaded onto the electroporation slides. The embryos were electroporated based on an assessment of several voltages and times, the optimum conditions being those used by Grabarek et al. [90]: 10 V voltage, 750 microseconds pulse length, 6 pulses, 100 millisecond interval between pulses, unipolar pulse. After electroporation the embryos were washed through HBS/BSA and cultured up to day 5 (as above). Development was scored at 24-hour intervals after electroporation (daily between 3 and 4 pm). At days 4 and 5 of culture some embryos were harvested for RT-PCR, others stained by immunocytochemistry or assessed for cell number and apoptosis by TUNEL assay.

### Rescue experiments

Glycerol stocks of purified recombinant Sox2-TAT fusion protein were generated as described previously [52]. The stocks were freshly diluted 1∶20 in KSOM media containing 0.5% Albumax II (Invitrogen) yielding final Sox2-TAT concentrations between 100 and 300 nM. *Sox2*-siRNA embryos were transferred and cultured in the above supplemented media 24 h after RNAi. Half the medium containing the Sox2-TAT protein was replenished every 24 h and embryos were cultured up to day 5. Rescue experiments were performed with *Sox2*-duplex-2 and *Sox2*-duplex-3 siRNA embryos.

### Immunostaining

Embryos were fixed in 4% paraformaldehyde (PFA, Sigma), washed through PBS-Tween with 4 mg/ml BSA (PBS/BSA), permeabilised with 1% Triton X-100 (Sigma) and washed through PBS/BSA. In order to decrease background staining, embryos were incubated for 10 min at room temperature in a solution of 2.6 mg/ml NH_4_Cl (Sigma) in PBS, and subsequently permeabilised and washed as above. They were then immersed in 1∶20 normal goat serum (NGS, Sigma) or normal donkey serum (NDS, Sigma) before incubation in the appropriate dilution of the primary antibody in PBS/BSA at 4°C overnight. They were then washed and transferred into the appropriate secondary antibody (Alexa-Fluor, Molecular Probes) at 1∶200 dilution in PBS/BSA and incubated for 1 hour in the dark at room temperature. After washing, embryos were mounted in DAPI Vectashield mountant (Vector Laboratories) and aspirated by capillary action into 0.2 µM diameter microcapillaries (Camlab), which were sealed in both ends. Primary antibodies were as follows: Sox2 mouse monoclonal (1∶50, R&D), Sox2 rabbit polyclonal (1∶500, Abcam), Sox2 rabbit polyclonal (1∶100, Chemicon), Oct4 mouse monoclonal (1∶250, BD Biosciences), Nanog goat IgG (1∶10, R&D systems), Yap rabbit polyclonal (1∶25, Cell Signaling), Cdx2 rabbit polyclonal (1∶500, raised in rabbits to an N-terminal KLH-linked peptide of mouse Cdx2 by Dr J. Collins, University of Southampton), Cdx2 mouse monoclonal IgG (1∶400, Biogenex, gift from Dr J. Draper, Toronto Hospital for Sick Children, Toronto), Eomesodermin rabbit polyclonal (1∶100, Orbigen), Fgfr2 mouse IgG (1∶100, Santa Cruz Biotechnology), Fgf4 goat polyclonal (1∶50, Santa Cruz Biotechnology), Occludin rabbit polyclonal (1∶100, Zymed), ZO1 mouse monoclonal IgG (1∶100, Zymed), Desmoplakin mouse monoclonal IgG (1∶10, gift from Prof. D. Garrod, University of Manchester), E-cadherin rat monoclonal IgG (1∶500, Sigma), Gata4 goat IgG (1∶200, Santa Cruz Biotechnology), Gata6 goat IgG (1∶200, R&D). Irrelevant antibodies of the same species/isotype as the primary antibodies replaced the primary antibodies, as negative controls: rabbit IgG (Vector laboratories); mouse IgG (Serotec); goat IgG (Santa Cruz Biotechnology); rat IgG (Santa Cruz Biotechnology).

### Confocal Microscopy

Embryos were viewed under a multi-photon scanning laser confocal microscope (MRC 1024, BioRad) or a Leica (TCS Sp2 AOBS) inverted confocal microscope at the Bioimaging Facility, University of Manchester. A Z-series of 2.5 µM optical sections was collected and analysed using Confocal Assistant software (version 4.02) or LCS Lite software.

### Trypan Blue viability assay

In order to assess the lethality of the *Sox2* siRNA phenotype, day 5 *Sox2* siRNA embryos were incubated in Trypan Blue (Sigma) solution, diluted 1∶10 in KSOM embryo culture medium at room temperature for 10 min before assessment by bright-field microscopy. Blue-stained cells were considered non-viable.

### TUNEL Assay

Control and *Sox2*-siRNA embryos were assessed for apoptosis using the TUNEL assay (Fluorescein conjugated In Situ Cell Death Detection Kit, Roche) as in Kamjoo et al. [58]. Day 4 and 5 embryos were fixed in 4% PFA, permeabilised in 0.5% Triton X-100, washed in PBS/PVP, and incubated for 1 hour at 37°C in the dark in TUNEL solution, a mixture of terminal deoxynucleotidyl transferase and fluorescein-conjugated dUTP in a ratio of 1∶9. Positive control embryos were incubated in DNase (Roche) for 20 minutes at 37°C before TUNEL labelling. Negative control embryos were incubated in fluorescein-conjugated dUTP only. After TUNEL-labelling, embryos were washed in 0.5% Triton X-100 and PBS/PVP, mounted with DAPI Vectashield and aspirated into microcapillary tubes, which were then examined by confocal microscopy.

### Assessment of apoptotic index and cell number

Apoptotic indices were calculated as number of apoptotic cells divided by total number of cells (DAPI stained nuclei) per embryo. Cell number per embryo was calculated by counting DAPI stained nuclei in confocal images produced by a compressed Z-series of 2.5 µM optical sections for each embryo by means of Confocal Assistant software (version 4.02).

### Statistical Analysis

SPSS software (version 11.5) was used to perform Independent Samples T-test for the apoptosis assessment. GraphPad Prism software (version 4) was used for the chi-square tests to monitor distribution of embryos among preimplantation developmental stages.

### RT-PCR

RNA was extracted from groups of 30 embryos by Dynabeads mRNA Direct Micro Kit (Dynal). cDNA was synthesized using Superscript II reverse transcriptase (Invitrogen) according to manufacturer's guidelines. The primers used (5′-3′) were:

***Sox2*** **(NM_011443)** CACAACTCGGAGATCAGCAA/CTCCGGGAAGCGTGTACTTA

***β-actin*** **(NM_007393)** AGCCATGTACGTAGCCATCC/CTCTCAGCTGTGGTGGTGAA

***Fgf4*** **(NM_010202)** TCGGTGTGCCTTTCTTTACC/ACCTTCATGGTAGGCGACAC

***Fgfr2*** **(NM_201601)** CACCAACTGCACCAATGAAC/GAATCGTCCCCTGAAGAACA

***Cdx2*** **(NM_007673)** AGGCTGAGCCATGAGGAGTA/CGAGGTCCATAATTCCACTCA

***Eomes*** **(NM_010136)** CCTGGTGGTGTTTTGTTGTG/TTTAATAGCACCGGGCACTC

***Tead4*** **(NM_011567)** AGCTAAGAACAAGGCCCTGC/TGCCAAAACCCTGAGATTGC

***Oct4*** **(NM_013633)** ATGGGGAAAGAAGCTCAGTG/CAAAATGATGAGTGACAGACAGG

***Nanog*** **(NM_028016)** CACCCACCCATGCTAGTCTT/ACCCTCAAACTCCTGGTCCT

***Sox1*** **(NM_009233)** TACAGCCCCATCTCCAACTC/TCCGACTTGACCAGAGATCC

***Sox14*** **(XM_907434)** TGCGCAATTTAGTTCCAGTG/ATGCCTGGGAAGAGGATGTA

***Sox21*** **(NM_177753)** TCCAAGCCTGTGGACCACGT/GAGCCATGCACATGAAGGAG

***Sox3*** **(NM_009237)** TCCGTGGTGAAGTCGGAG/GCCCTGGTAGTGCTGGTG

***Sox15*** **(NM_009235)** GGCGTAAGAGCAAAAACTCG/TGGGATCACTCTGAGGGAAG

***Atp1b1*** **(NM_009721)** CAGATTCCCCAGATCCAGAA/CTGCACACCTTCCTCTCTCC

***Gata3*** **(NM_008091)** GTGGTCACACTCGGATTCCT/GCAAAAAGGAGGGTTTAGGG

***Gata4*** **(NM_008092)** TCTCACTATGGGCACAGCAG/CGAGCAGGAATTTGAAGAGG

***Gata6*** **(NM_010258)** GAGCTGGTGCTACCAAGAGG/TGCAAAAGCCCATCTCTTCT

Forty-cycle PCR amplification was performed using thermal cycler Mastercycler Gradient (Eppendorf). Amplicons, each amplified from 50 ng cDNA, were run by electrophoresis alongside a 100 bp DNA ladder (Invitrogen) or Hyperladder IV (Bioline) on 2% agarose gels, at 100 V and 150 mA for 1 hour. Mouse genomic DNA (Bioline) was used as positive control.

### Sequencing

PCR product bands of the correct size were confirmed to contain the expected gene product by DNA sequencing. Bands were excised from the agarose gels and DNA extracted and purified using QIAquick Gel-Extraction-Kit (QIAGEN) which were then sequenced (in the Sequencing Unit) using an ABI Prism 377 sequencer (Applied Biosystems). Chromas software was used to read the base compositions, which were copied into the NCBI BLAST database (http://www.ncbi.nih.gov/BLAST/) to identify the sequences.

### Western blotting

Validation of Sox2 antibody specificity was performed by Western blotting using 2×10^7^ mouse ES cells (line R1)/ml of lysis buffer (50 mM Tris-Cl, 150 mM NaCl, 0.02% Na azide, 1% Triton X-100) with 1 protease inhibitor cocktail tablet (Complete Mini, Roche)/10 ml lysis buffer. A protein assay was performed using the bicinchoninic acid (BCA) Kit (Pierce), following the Microplate procedure, according to manufacturers' instructions. Twenty five µl lysates samples (50 µg of protein) were resolved by a 4–12% NuPAGE Bis-Tris gel according to manufacturer's recommendations (Invitrogen). 10 µl of BioRad kaleidoscope Multi-Coloured Standard markers were run in parallel at 200 V, 110 mA for 50 minutes. Samples were transferred to a PVDF membrane (Amersham Biosciences), which was blocked in 5% w/v dried milk powder (Marvel) in 0.1% PBS-Tween, incubated with Sox2 antibody (1∶1000, Abcam) in 5% w/v dried milk powder (Marvel) at 4°C overnight and then with a peroxidase-labelled anti-rabbit IgG (1∶3000, DAKO) in 5% w/v dried milk powder (Marvel) for 1 hour. The enhanced chemiluminescence (ECL) detection system (Amersham Biosciences) was used to visualize the protein bands.

## Supporting Information

Figure S1 (A) Sox2 antibody positive controls: pluripotent mES cells (Sox2-ES) stained for Sox2 (Abcam) in nuclei with some cytoplasmic staining, as well as neurally differentiated E14 cells (Sox2-N) with mainly nuclear Sox2 protein localisation and negative immunological controls (rabbit IgG isotype control and 2° Ab only control) for Sox2 embryo staining. Bars: 100 μm. (B) Western blot to validate specificity of the Sox2 (Abcam) antibody; 50μg mES protein loaded. Lane 1: 2o antibody only; 2: Sox2 (37kDa).
(1.24 MB DOC)

Figure S2 Development of MF1xMF1 embryos after RNAi. Sox2-duplex-1-siRNA embryos (hatched, N = 30) were compared with incubator-control (grey, N = 20), FFL-siRNA (black, N = 27) and bench-control embryos (white, N = 22). On day 5, while control embryos formed blastocysts from 80% to 92.6%, only 3.3% of the Sox2-siRNA embryos formed blastocysts, with 96.7% arresting at the morula stage. On day 5, chi-square tests revealed significant differences (p<0.0001) between the % of Sox2-siRNA morulae and all control morulae, as well as between the % of Sox2-siRNA blastocysts and all control blastocysts.
(0.22 MB DOC)

Figure S3 Immunostaining of day 4 and day 5 incubator ctrl (untreated) and Sox2-duplex-3-siRNA embryos, with different Sox2 (Chemicon) and Cdx2 (Biogenex) antibodies than the ones presented in Figure 4. This confirms the expression pattern described in Figures 3 and 4 for both markers, as well as absence of Sox2 and Cdx2 proteins after Sox2 RNAi. The images are single optical sections from confocal Z-series. At least 10 embryos were stained for each antigen and representative embryos (not Sox2 siRNA ‘escapees’) are shown. Bars: 50 μm.
(1.54 MB DOC)

Figure S4 RT-PCR for Sox2, Fgfr2, Fgf4, Cdx2, Eomes, Tead4, Oct4, Nanog, Sox1, Sox3, Sox14, Sox15, Sox21 and Beta-actin (40 cycles) on day 4: incubator-control embryos (lanes 1 and 3); Sox2-duplex-1-siRNA embryos (lane 2); Sox2-duplex-3-siRNA embryos (lane 4). In the absence of Sox2 transcripts after siRNA, a clear reduction of Fgfr2, Ffg4, Cdx2, Eomes and Tead4 transcripts in Sox2-siRNA embryos was observed. Oct4 and Nanog transcripts were unaffected in Sox2 knock-down morulae compared to incubator-control morulae. Sox1, Sox3, Sox14, Sox15 and Sox21 transcripts were not expressed in any of the control or Sox2 knock-down morulae. Beta actin transcripts were detected in all embryos.
(0.70 MB DOC)

Figure S5 Immunofluorescence confirmation of transferred Sox2 after initiation of the rescue experiment using the Sox2-TAT protein. Sox2 siRNA R embryos were immunostained with Sox2 6h, 12h and 18h after the first addition of Sox2-TAT protein, to assess Sox2 protein recovery efficiency. Gradual expression of Sox2 protein was confirmed, with signs of possible endocytic uptake of the protein, as indicated by patchy expression of Sox2.
(1.07 MB DOC)

Figure S6 Immunological controls of embryo staining presented in Figures 3 and 4; the upper panel (A to K) shows controls for dual staining, the lower panel (L to S) illustrates controls for staining with single antibodies. The images are single optical sections from confocal Z-series. In all cases, the controls were negative. A: anti-Sox2 1o + goat anti-mouse IgG (2o to Oct4, Cdx2Biogenex, Fgfr2, ZO1, Desmoplakin); B: anti-Oct4 1o + goat anti-rabbit IgG (2o to Sox2); C: anti-Cdx2Biogenex 1o + goat anti-rabbit IgG (2o to Sox2); D: anti-Fgfr2 1o + goat anti-rabbit IgG (2o to Sox2); E: anti-ZO1 1o + goat anti-rabbit IgG (2o to Sox2); F: anti-Desmoplakin 1o + goat anti-rabbit IgG (2o to Sox2); G: anti-Sox2 1o + donkey anti-goat IgG (2o to Nanog and Fgf4); H: anti-Nanog 1o + donkey anti-rabbit IgG (2o to Sox2); I: anti-Fgf4 1o + donkey anti-rabbit IgG (2o to Sox2); J: anti-E-cadherin 1o + goat anti-rabbit IgG (2o to Sox2); K: anti-Sox2 1o + goat anti-rat IgG (2o to E-cadherin); L: Rabbit IgG isotype control to Sox2, Cdx2Jane Collins, Eomes, Occludin 1o antibodies; M: Mouse IgG isotype control to Oct4, Cdx2Biogenex, Fgfr2, ZO1, Desmoplakin 1o antibodies; N: Goat IgG isotype control to Nanog, Fgf4 1o antibodies; O: rat IgG isotype control to E-cadherin 1o antibody; P: Goat anti-rabbit IgG 2o antibody only control to Sox2, Cdx2Jane Collins, Eomes, Occludin; Q: Goat anti-mouse IgG 2o antibody only control to Oct4, Cdx2Biogenex, Fgfr2, ZO1, Desmoplakin; R: Donkey anti-goat IgG 2o antibody only control to Nanog, Fgf4; S: Goat anti-rat IgM 2o antibody only control to E-cadherin; T: Goat anti-rabbit IgG 2o antibody only control to Yap; U: Donkey anti-goat IgG 2o antibody only control to Gata4, Gata6; V: Goat IgG isotype control to Gata4, Gata6. Bars: 50 μm.
(1.16 MB DOC)
