# Supplementary figures and images for: Sox2 Is Essential for Formation of Trophectoderm in the Preimplantation Embryo
Source: PLoS One. 2010 Nov 12;5(11):e13952. doi: 10.1371/journal.pone.0013952 (PMC2980489; doi:10.1371/journal.pone.0013952)

**Figure S1**

**A**

**
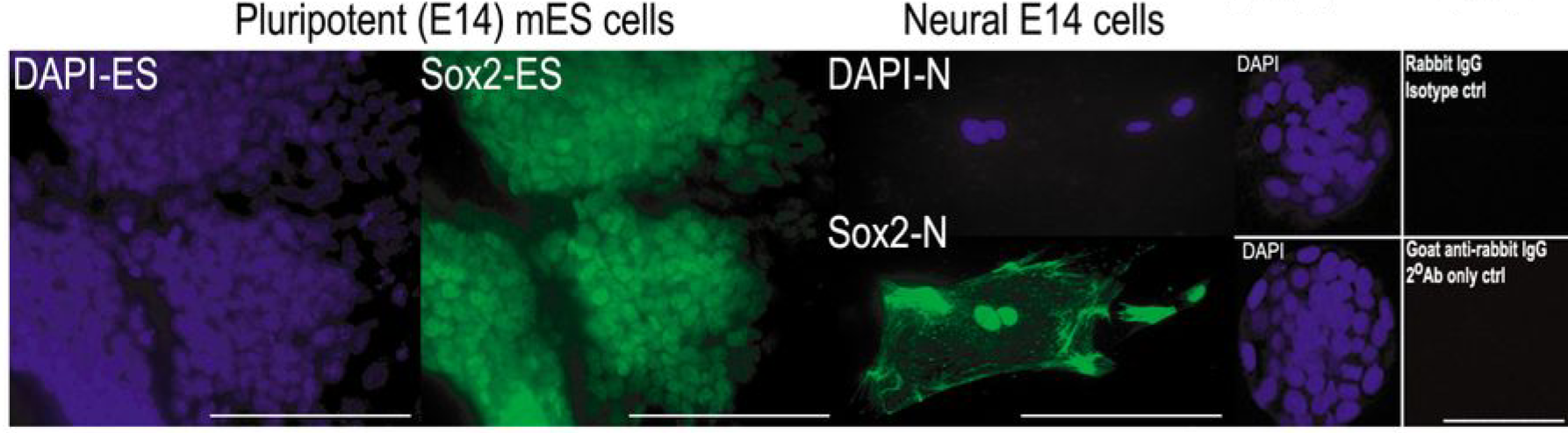
**

**B**


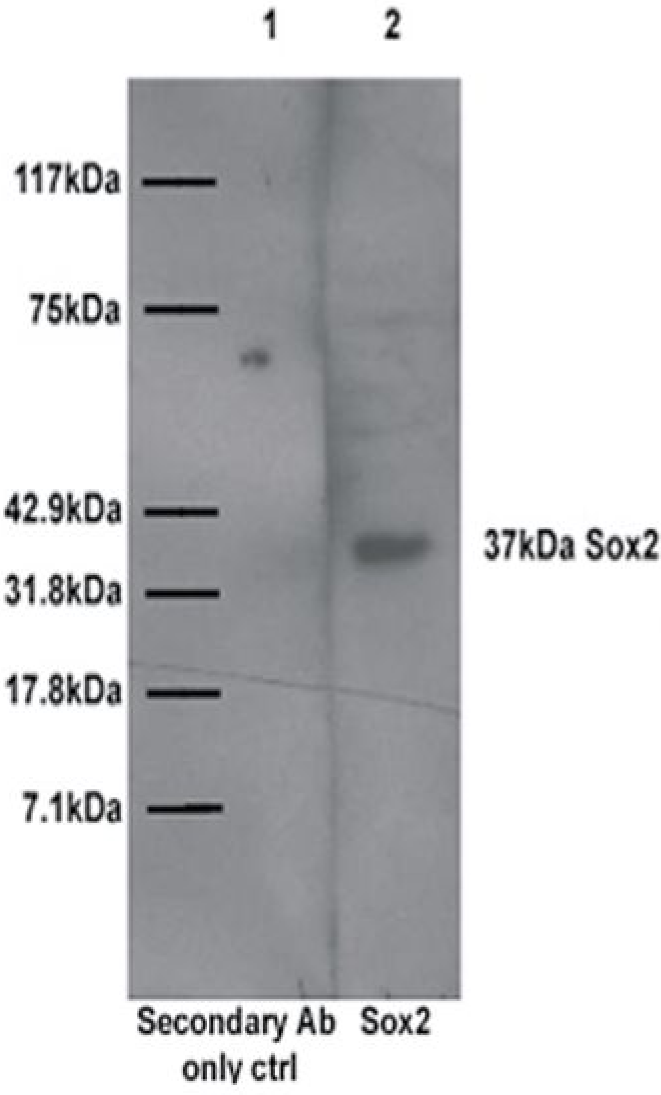

Supplement: Figure S1 — (A) Sox2 antibody positive controls: pluripotent mES cells (Sox2-ES) stained for Sox2 (Abcam) in nuclei with some cytoplasmic staining, as well as neurally differentiated E14 cells (Sox2-N) with mainly nuclear Sox2 protein localisation and negative immunological controls (rabbit IgG isotype control and 2° Ab only control) for Sox2 embryo staining. Bars: 100 μm. (B) Western blot to validate specificity of the Sox2 (Abcam) antibody; 50μg mES protein loaded. Lane 1: 2o antibody only; 2: Sox2 (37kDa). (1.24 MB DOC) [file pone.0013952.s001.doc]

**Figure S2**


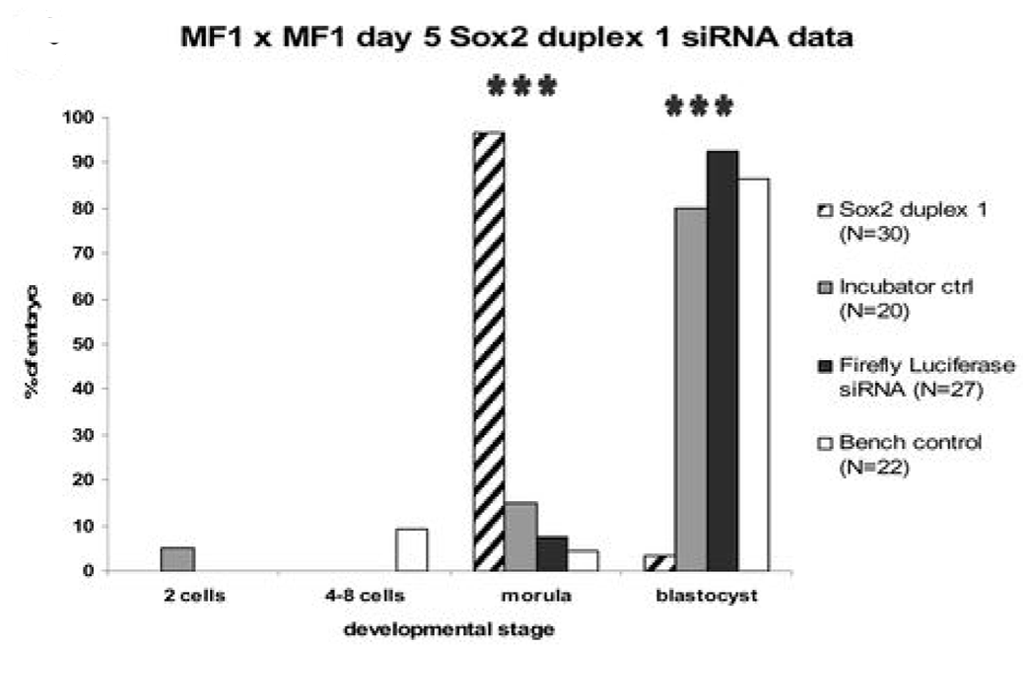

Supplement: Figure S2 — Development of MF1xMF1 embryos after RNAi. Sox2-duplex-1-siRNA embryos (hatched, N = 30) were compared with incubator-control (grey, N = 20), FFL-siRNA (black, N = 27) and bench-control embryos (white, N = 22). On day 5, while control embryos formed blastocysts from 80% to 92.6%, only 3.3% of the Sox2-siRNA embryos formed blastocysts, with 96.7% arresting at the morula stage. On day 5, chi-square tests revealed significant differences (p<0.0001) between the % of Sox2-siRNA morulae and all control morulae, as well as between the % of Sox2-siRNA blastocysts and all control blastocysts. (0.22 MB DOC) [file pone.0013952.s002.doc]

**Figure S3**


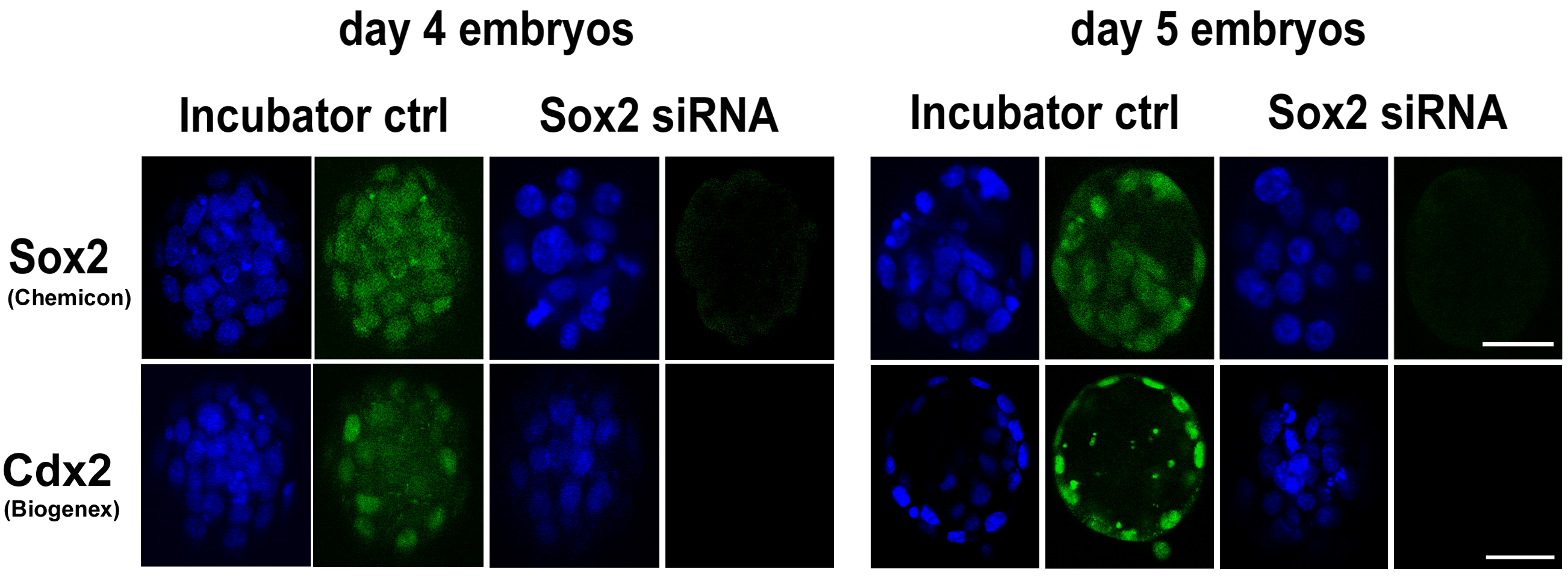

Supplement: Figure S3 — Immunostaining of day 4 and day 5 incubator ctrl (untreated) and Sox2-duplex-3-siRNA embryos, with different Sox2 (Chemicon) and Cdx2 (Biogenex) antibodies than the ones presented in Figure 4. This confirms the expression pattern described in Figures 3 and 4 for both markers, as well as absence of Sox2 and Cdx2 proteins after Sox2 RNAi. The images are single optical sections from confocal Z-series. At least 10 embryos were stained for each antigen and representative embryos (not Sox2 siRNA ‘escapees’) are shown. Bars: 50 μm. (1.54 MB DOC) [file pone.0013952.s003.doc]

**Figure S4**


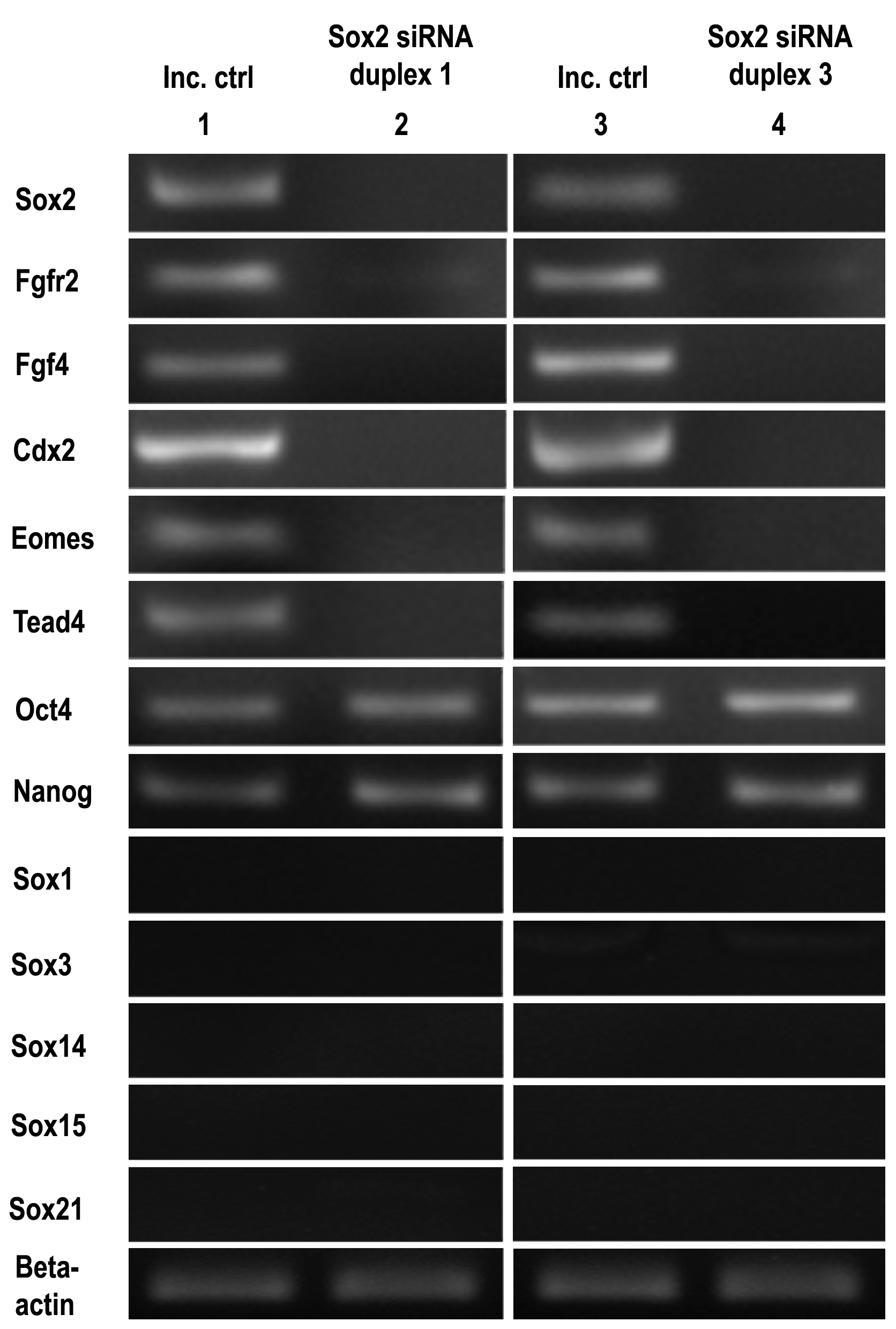

Supplement: Figure S4 — RT-PCR for Sox2, Fgfr2, Fgf4, Cdx2, Eomes, Tead4, Oct4, Nanog, Sox1, Sox3, Sox14, Sox15, Sox21 and Beta-actin (40 cycles) on day 4: incubator-control embryos (lanes 1 and 3); Sox2-duplex-1-siRNA embryos (lane 2); Sox2-duplex-3-siRNA embryos (lane 4). In the absence of Sox2 transcripts after siRNA, a clear reduction of Fgfr2, Ffg4, Cdx2, Eomes and Tead4 transcripts in Sox2-siRNA embryos was observed. Oct4 and Nanog transcripts were unaffected in Sox2 knock-down morulae compared to incubator-control morulae. Sox1, Sox3, Sox14, Sox15 and Sox21 transcripts were not expressed in any of the control or Sox2 knock-down morulae. Beta actin transcripts were detected in all embryos. (0.70 MB DOC) [file pone.0013952.s004.doc]

**Figure S5**

**
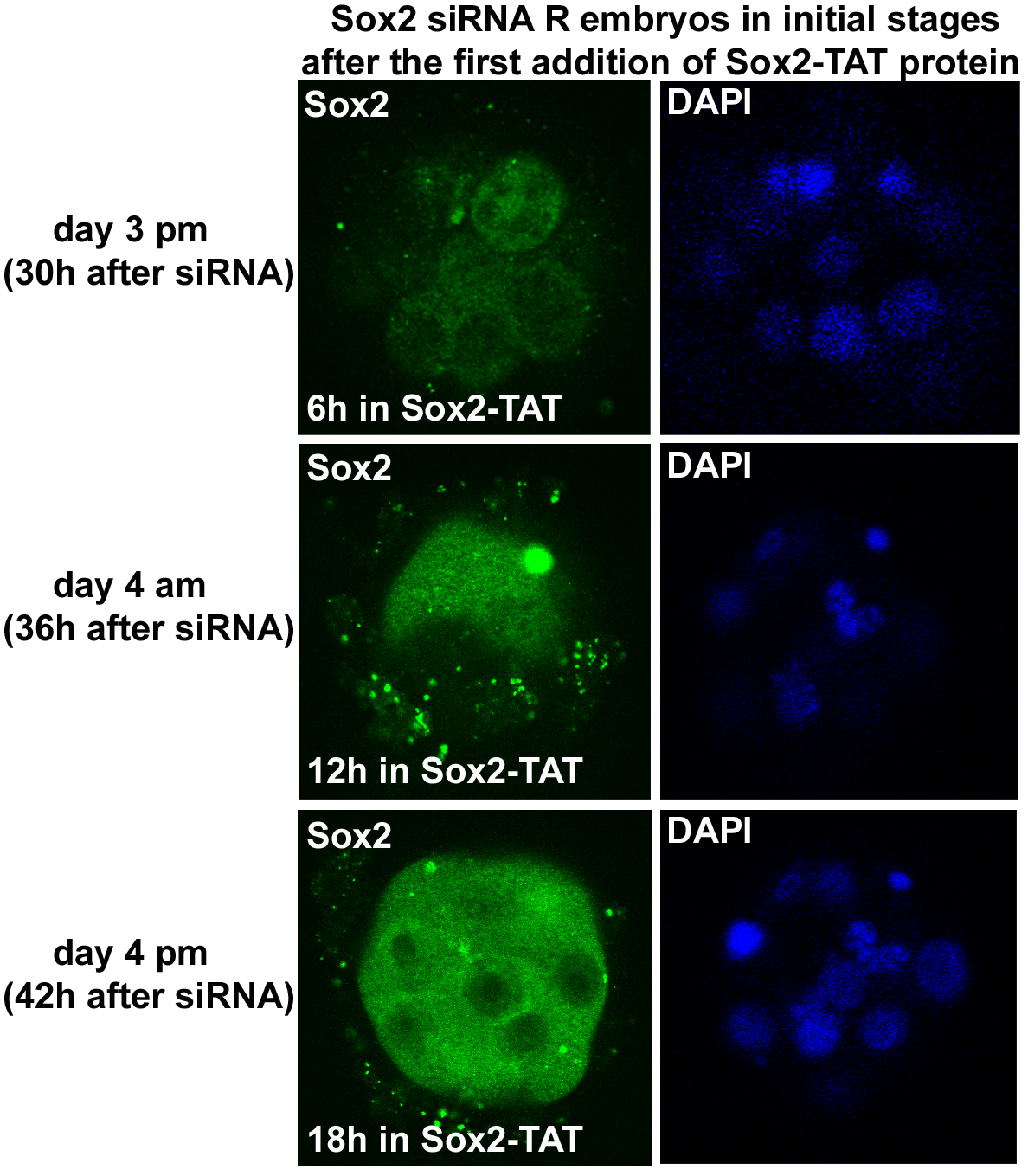
**

Supplement: Figure S5 — Immunofluorescence confirmation of transferred Sox2 after initiation of the rescue experiment using the Sox2-TAT protein. Sox2 siRNA R embryos were immunostained with Sox2 6h, 12h and 18h after the first addition of Sox2-TAT protein, to assess Sox2 protein recovery efficiency. Gradual expression of Sox2 protein was confirmed, with signs of possible endocytic uptake of the protein, as indicated by patchy expression of Sox2. (1.07 MB DOC) [file pone.0013952.s005.doc]

**Figure S6**

**
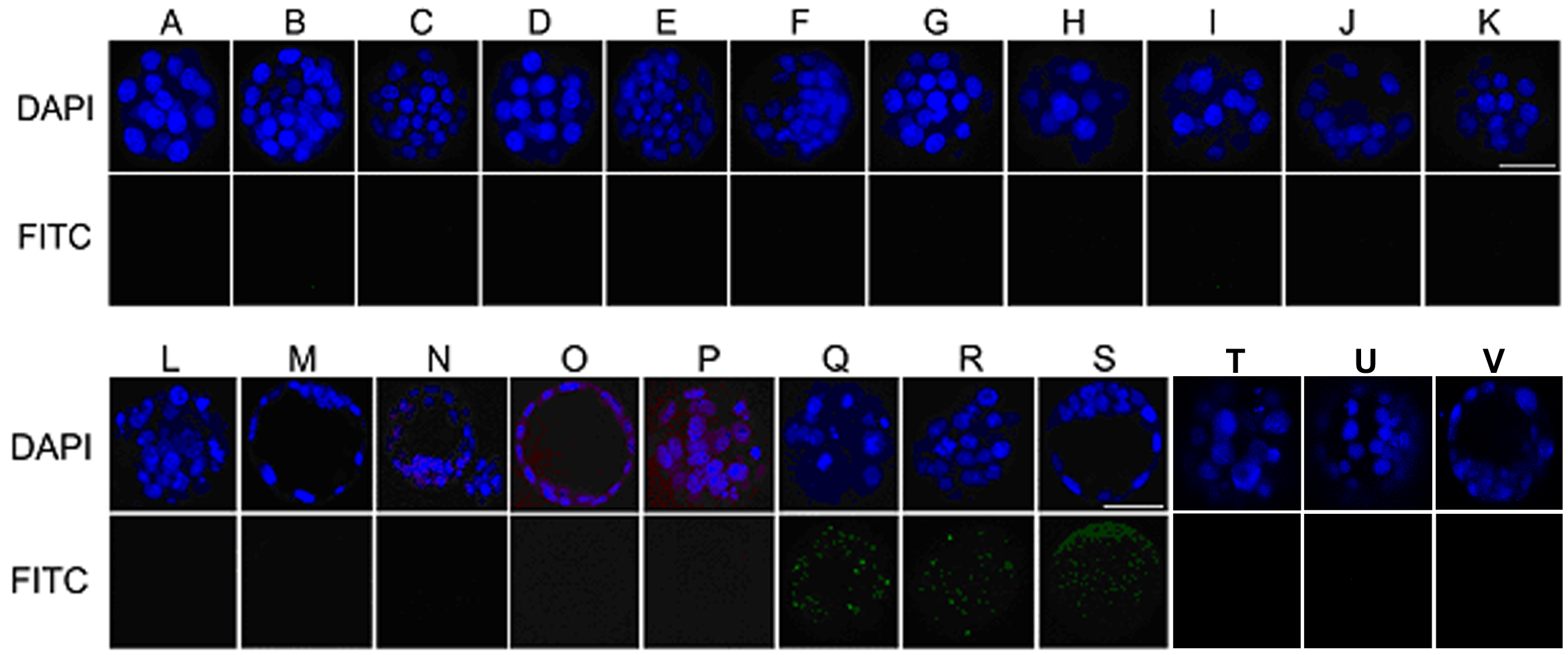
**

Supplement: Figure S6 — Immunological controls of embryo staining presented in Figures 3 and 4; the upper panel (A to K) shows controls for dual staining, the lower panel (L to S) illustrates controls for staining with single antibodies. The images are single optical sections from confocal Z-series. In all cases, the controls were negative. A: anti-Sox2 1o + goat anti-mouse IgG (2o to Oct4, Cdx2Biogenex, Fgfr2, ZO1, Desmoplakin); B: anti-Oct4 1o + goat anti-rabbit IgG (2o to Sox2); C: anti-Cdx2Biogenex 1o + goat anti-rabbit IgG (2o to Sox2); D: anti-Fgfr2 1o + goat anti-rabbit IgG (2o to Sox2); E: anti-ZO1 1o + goat anti-rabbit IgG (2o to Sox2); F: anti-Desmoplakin 1o + goat anti-rabbit IgG (2o to Sox2); G: anti-Sox2 1o + donkey anti-goat IgG (2o to Nanog and Fgf4); H: anti-Nanog 1o + donkey anti-rabbit IgG (2o to Sox2); I: anti-Fgf4 1o + donkey anti-rabbit IgG (2o to Sox2); J: anti-E-cadherin 1o + goat anti-rabbit IgG (2o to Sox2); K: anti-Sox2 1o + goat anti-rat IgG (2o to E-cadherin); L: Rabbit IgG isotype control to Sox2, Cdx2Jane Collins, Eomes, Occludin 1o antibodies; M: Mouse IgG isotype control to Oct4, Cdx2Biogenex, Fgfr2, ZO1, Desmoplakin 1o antibodies; N: Goat IgG isotype control to Nanog, Fgf4 1o antibodies; O: rat IgG isotype control to E-cadherin 1o antibody; P: Goat anti-rabbit IgG 2o antibody only control to Sox2, Cdx2Jane Collins, Eomes, Occludin; Q: Goat anti-mouse IgG 2o antibody only control to Oct4, Cdx2Biogenex, Fgfr2, ZO1, Desmoplakin; R: Donkey anti-goat IgG 2o antibody only control to Nanog, Fgf4; S: Goat anti-rat IgM 2o antibody only control to E-cadherin; T: Goat anti-rabbit IgG 2o antibody only control to Yap; U: Donkey anti-goat IgG 2o antibody only control to Gata4, Gata6; V: Goat IgG isotype control to Gata4, Gata6. Bars: 50 μm. (1.16 MB DOC) [file pone.0013952.s006.doc]
